# Factors associated with school dropout and sexual and reproductive health: a cross-sectional analysis among out-of-school girls in western Kenya

**DOI:** 10.1136/bmjph-2024-001528

**Published:** 2025-03-04

**Authors:** Susan Nungo, Anna Maria van Eijk, Linda Mason, Elizabeth Nyothach, Benard Asuke, Philip Spinhoven, David Obor, Christine Khaggayi, Daniel Kwaro, Penelope A Phillips-Howard, Garazi Zulaika

**Affiliations:** 1 Liverpool School of Tropical Medicine, Liverpool, UK; 2 Kenya Medical Research Institute, Nairobi, Kenya; 3 Leiden University, Leiden, The Netherlands; 4 Safe Water and AIDS Project, Kisumu, Kenya

**Keywords:** Epidemiology, Community Health, Education, Female, Sociodemographic Factors

## Abstract

**Introduction:**

Out-of-school girls are at higher risk of sexual and reproductive health (SRH) harms. Schools provide a protective environment for adolescents and lessen their exposure to such risks. This paper explores factors associated with school dropout, sexual activity, marriage and pregnancy among out-of-school girls in western Kenya.

**Methods:**

Eligible adolescents were systematically recruited from area households in Siaya County. Generalised linear models were fit to obtain adjusted ORs (aOR) and 95% CIs of key covariates against individual outcomes. Factors with p values <0.1 in the univariate analysis were added to a multivariable model using backward stepwise regression techniques, and factors significant at p<0.05 were retained in the final adjusted models. Models were bootstrapped at 1000 replications to validate factor selection.

**Results:**

Of the 915 girls enrolled (mean 18.3 years, SD: 1.3), 2.1% had never attended school. Of those who started school, 34.6% dropped out during primary education. Reasons for dropout included marriage, pregnancy and needing childcare (42.5%), financial reasons (eg, lack of school fees, needing to work, 42.5%), lack of interest (5.6%), illness (3.0%), failing school (2.2%) and other factors (4.1%). Reaching menarche prior to age 13 (aOR 1.50, 95% CI 1.00 to 2.23, p=0.048), experiencing physical violence (aOR 1.48, 95% CI 1.01 to 2.17, p=0.042) or sexual partner violence (aOR 2.16, 95% CI 1.08 to 4.34, p=0.030) were associated with not completing primary school. Experiencing sexual harassment (aOR 2.20, 95% CI 1.35 to 3.58, p=0.002) or needing to engage in transactional sex (aOR 1.74, 95% CI 1.20 to 2.51, p=0.003) were associated with being sexually active. Low socioeconomic status (aOR 1.98, 95% CI 1.36 to 2.90, p<0.001), having an older partner (aOR 1.65, 95% CI 1.10 to 2.47, p=0.016) and higher parity (aOR 2.56, 95% CI 1.42 to 4.62, p=0.002) were associated with being married or cohabiting with a partner. Girls identified provision of school fees and schooling items (67.9%) as the primary solution to resuming school; obtaining counselling, mentorship and support services (22.2%) for their general health; and provision of menstrual products (24.2%) for daily challenges.

**Conclusions:**

Out-of-school girls in western Kenya face numerous SRH challenges related to menstruation, sexual and physical violence, and poverty. Social and financial support and interventions for school re-entry are warranted for this neglected population. National policies and multisectoral strategies to support adolescent girls’ education and health should be prioritised, enforced and monitored for impact.

**WHAT IS ALREADY KNOWN ON THIS TOPIC**

- Girls who are out of school are exposed to numerous risks which impede resuming school and which have detrimental effects on their sexual and reproductive health (SRH) and well-being.
- Approximately 1.8 million children and adolescents in Kenya aged 6–17 years were out of school in 2021.

**WHAT THIS STUDY ADDS**

- Girls who reached menarche at younger ages and those who were exposed to physical violence or sexual partner violence were more likely to drop out of school at an earlier age.
- Sexual harassment was prevalent, with girls who experienced it more likely to report sexual activity in the past 6 months.
- Poverty, age-discordant relationships and early pregnancies were associated with early marriage among adolescents in western Kenya.
- Most girls wished to return to school, voicing needing assistance with school feels to do so. When asked about their health and general daily challenges, girls sought counselling and menstrual products.

**HOW THIS STUDY MIGHT AFFECT RESEARCH, PRACTICE OR POLICY**

- Our findings indicate that out-of-school adolescent girls face frequent harassment for sex and are at high risk of early pregnancy, marriage and related SRH harms, which compound risks and hinder them from resuming school.
- Gender and age-sensitive school interventions are needed to prevent school dropout and support older girls’ resumption of schooling to safeguard their educational and health outcomes.

## Introduction

 Adolescence is a life stage characterised by puberty, sexual initiation and risk-taking.^1^ In low-income and middle-income countries, girls in this life stage are at heightened risk of adverse events such as early marriage, female genital mutilation, child labour, sexual harassment and exploitation, domestic and physical abuse, and sexual and reproductive harms such as early pregnancy and sexually transmitted infections (STIs).^1^ Research has highlighted that schools provide a protective environment, especially for adolescent girls who are most vulnerable to these challenges,^2^,^4^ although strengthened effort is required to ensure the continued safety of all learners.^5 6^ A positive schooling environment protects against risky behaviours such as alcohol, drug and substance use,^7 8^ and girls’ connection with teachers has been associated with improved mental health.^9^ Girls’ education underpins Sustainable Development Goals 3 (Good Health and Wellbeing) and 4 (Quality Education), making it critical to attaining these and other targets.^10 11^

Evidence supports that higher school completion among girls reduces their risk of HIV/AIDS, intimate partner violence (IPV), child marriage and early childbearing.^12^,^15^ Young childbearing has additional direct negative impacts on girls’ health and well-being and on the health of their children.^10^ Research indicates that one in five under-5 child deaths would be averted with maternal completion of secondary education.^10^ Moreover, incomes and living standards have been found to be directly proportional to the level of education attained by girls.^10 11^ Post secondary education promises better chances in the labour market and increases incomes compared with primary level or no education at all.^11^ It also improves girls’ agency, decision-making capabilities and social capital.^10^

In sub-Saharan Africa (SSA), one in five primary school-aged children, one in three adolescents in lower secondary and one in two in upper secondary school are out of school.^16^ Despite significant improvements in access to education in the region, progress has slowed, and gender disparities in access continue to be pervasive, with girl children more at risk of being denied schooling.^1^,^416 17^ It is estimated that, as of 2018, nine million girls in SSA aged 6–11 years will never attend school.^18^ Gender disparities in the region are notable, with models indicating that 23% of girls aged 6–11 years who started primary school drop out, compared with 19% of boys.^16 18^ These disparities become greater at secondary school level, with 37% of girls and 32% of boys being out of school by lower secondary, and up to 61% of girls vs 55% of boys out of school by upper secondary.^18^

In Kenya, interventions have been implemented to improve access to education. Interventions rolled out to improve school enrolment, re-enrolment and school retention include cash transfer programmes, provision of menstrual products, supply of education materials, teacher trainings, community mobilisation and policy development.^19^,^23^ On a national level, the Constitution of Kenya declares that education is compulsory and stipulates that every child has a basic human right to free education.^24^ However, as of 2021, about 1.8 million children and adolescents in Kenya aged 6–17 years were out of school.^25^ Various recent strategic frameworks have been developed in response, such as the National Education Strategic Plan 2023–2027^26^ and the Commitment Plan to End the ‘Triple Threat’ 2023–2030.^27^ These national-level plans target reducing new HIV infections, mistimed pregnancies, and sexual and gender-based violence in adolescents and young people, and commit the country to increasing the proportion of primary school pupils transitioning to secondary school, and enforcing and fully implementing Kenya’s school re-entry policy for adolescent mothers.

Out-of-school girls have unmet health and socioeconomic needs. Research in partnership with girls and community stakeholders can uncover these problems and identify potential solutions. Few studies have examined the characteristics of out-of-school girls and the challenges they face. This paper presents data from a community-based cross-sectional survey that examined the individual and household level characteristics of out-of-school girls in western Kenya and their association with school dropout, sexual activity, early marriage and adolescent pregnancy. It also reports on the support girls themselves request as necessary to improve their lives.

## Methods

### Study setting

The study took place in Alego Usonga, Gem and Rarieda subcounties of Siaya County, located within the Lake Victoria basin in western Kenya. Covering about 2530 km^2^, the county holds a population of about 993 000, most of whom depend on fishing and subsistence farming for their livelihoods.^28^ In 2017, 4% of primary school students and 37% of secondary school students in Siaya County were out of school.^28^ The 2022 National Demographic and Health Survey reported that in Siaya County, for the general population of adolescent girls, age at first sex was 16.3 years and 18.4% of those aged 15–19 had ever had a live birth.^29^ The Human Development Index measure of literacy, health and well-being in the county stands at 0.445, lower than the national average of 0.532.^28^ Young people in the region face acute health challenges, including high rates of HIV and STIs, teenage pregnancy, gender-based violence and rising mental health disorders.^28^

### Study design

This cross-sectional study was nested within the Kenya Medical Research Institute’s (KEMRI) Health and Demographic Surveillance System (HDSS). Participants were recruited from a biannual HDSS census that collected information on all household members living in the area through house-to-house surveys. Due to out-of-school girls being hard to reach, local community health workers and other community informants also helped to identify school-aged girls who were not attending school in their catchment areas. Girls were eligible if they were out of school for more than three months, between the ages of 10–19 years, had begun menstruating, were area residents and had no disability precluding participation. Girls were no longer eligible if they had re-enrolled in school or moved out of the study area since being identified in the prior HDSS census. All eligible girls were invited to participate.

All participants over the age of 18 years provided written informed consent to participate. Written assent and guardian consent was obtained for non-emancipated minors. In accordance with Kenyan guidelines, parental consent was not required for girls under 18 years who were emancipated or assumed adult responsibilities such as being married or having entered motherhood. Participants were informed of their right to refuse or withdraw from the study at any time.

### Data collection, processing and analysis

Participant data were collected through validated sociodemographic survey questionnaires in English and Dholuo on electronic Android tablets, using Open Data Kit software (online supplemental file S1, Participant Survey). Surveys were self-administered when possible and were interviewer-administered if a participant could not complete the survey on her own. HDSS household data on socioeconomic status (SES) was integrated with participant data. All personal identifying data were anonymised and number-coded at the source, and storage files were encrypted.

The main outcome measures of the study comprised 1) education level attained before dropout, (2) reason for dropout, (3) history of sex and current sexual activity, (4) marriage and (5) adolescent pregnancy. Education level reached prior to dropout was recategorised into (1) minimum to none or (2) completed primary school and/or initiated secondary school education. Reasons for school dropout were categorised into (1) marriage, pregnancy and childcare or (2) lack of school fees and need to work. The history of sexual activity was constructed from reporting engaging in sex (consensual, forced or coerced) and/or reporting current or past pregnancy. Participants were asked open-ended questions to identify solutions and resources they felt they needed to allay their general and health challenges and to resume schooling, which were then grouped into nine distinct categories for analysis.

The outcome variables were analysed against a set of covariates featuring individual characteristics (age, sexual activity, pregnancy history, parity, and condom and contraceptive use), family and partner characteristics (partner age, marital status, household composition, and childcare), household characteristics (SES, employment), menstrual health and hygiene (age at menarche, menstrual product use, period severity and duration, and menstruation impact on daily activities), general health and wellness (use of healthcare services, being happy at home, support from friends and family) and experiences of sexual harassment (indecent touching) and violence (IPV, any violence).

Survey data were analysed using STATA V.17.0. Generalised linear models were fit to obtain ORs and 95% CIs of key covariates against individual outcome measures. SES and age were included as *a priori* selected correlates and always presented in the univariate analysis. For each outcome variable, risk factors with a p value of <0.1 in the univariate analysis were added to multivariable models using backward stepwise regression techniques for model fit. Factors that were significant at p<0.05 were retained in the final multivariable model, and their adjusted ORs (aORs) and CIs are presented. Variance inflation factor (VIF) values were checked in models where collinearity between covariates could be suspected. If an explanatory variable was highly collinear with an outcome of interest, the explanatory variable was dropped from the model. Explanatory variables that were collinear (VIF>2.0) were investigated separately. To validate the stepwise regression results, the multivariable models with forced entry of all significant risk variables at p<0.10 were bootstrapped at 1000 replications (online supplemental table S1).

## Results

### General characteristics

Between 1 November 2021 and 24 January 2023, over 2800 households were approached. Study counsellors identified 916 eligible out-of-school girls, of whom 915 consented to participate in the study (median age 18.6, 17.5–19.4, online supplemental table S2). Most girls reported not living with their biological parents (73.8%). The mean number of years in school was 9.5 years (SD:2.5), with only 2.1% of girls reporting never having been to school at all (n=19). Among those who had been to school, 34.6% reported not completing primary school, and 65.4% did not progress to or dropped out during secondary school. Among all girls who had attended school, 88.3% had wished to continue school at the time of dropout and 55.7% reported still wanting to resume school. Reasons for dropping out included: pregnancy, marriage and/or childcare responsibilities (42.5%); financial challenges (ie, lack of school fees or needing to work) (42.5%); lack of interest (5.6%); illness (3.0%); failing at school (2.2%) and unspecified (4.1%). Of those who dropped out due to pregnancy, 70.4% reported being unable to return to school due to lack of childcare for their babies, teasing about having been pregnant or being refused re-entry to school by either their parents or the school; the remaining 29.6% reported financial reasons as the main reason for not resuming school.

A prior pregnancy was reported by 77.3% of girls; 17.6% of all girls self-reported being pregnant at the time of the survey. Most pregnancies were unintended (82.6%), and 28 girls (4.1%) reported attempting to terminate a prior pregnancy. At the time of the survey, 65.6% of girls had a child they were taking care of at home, with 97.8% of them indicating the child was their own (online supplemental tableS2). Among all girls, 34.3% were already married, cohabiting or widowed, reporting a mean age of 17.2 years (SD:1.1) at first marriage. Family planning use was reported by 31.2% of all girls, with the majority of those using contraceptives having previously been pregnant (90.9%). Hormonal contraceptives were used by 36.6% of girls who had experienced a prior pregnancy, and 12.5% of girls who had never been pregnant. Girls who used family planning predominantly chose hormonal contraceptive methods (implant 52.1%, injection 37.6%, birth control pills 5.5%), with only 5.5% reporting use of barrier methods and/or fertility awareness.

Sexual debut was reported by 85.1% of girls (mean age at first sex 16.0 years (SD: 1.7)). Of girls reporting prior sexual activity, 39.5% reported their first sex was undesired, 8% reported that their first sex partner was someone they did not know and 12 girls (1.7%) reported their first sex was with a relative. Transactional sex (ie, having sex in exchange for money or goods) was reported by 38.7% of girls (online supplemental table S2). Of girls who were currently sexually active (60.5% in the past 6 months), 29.4% of them reported that their current sexual partner was older than them by five or more years. IPV was reported by 15.7% of girls in a sexual relationship, with nearly half (7.6%, n=69) reporting more than one form of IPV: 86 girls (9.4%) reported experiencing physical violence, 52 girls (5.7%) reported sexual violence and 93 girls (10.2%) reported emotional violence from their intimate partner.

Girls’ mean age at menarche was 14.1 years (SD:1.6); 15.8% of girls reached menarche early (prior to age 13). When asked about menstrual hygiene product use, 87.9% reported using disposable sanitary pads during their last period. However, 50.5% of all respondents reported using a mix of menstrual products, with the majority reporting having also used cloth, blanket or rags (33.1%), cotton wool (19.0%) or reusable pads (18.4%), and a small number reporting using paper or cardboard (2.6%), menstrual cups (2.3%) or grass and leaves (1.6%) during their last period. Menstrual periods of long duration (7+ days) were reported by 17.9% girls, and 27.5% of girls reported that their bleeding was heavy. Period pain or cramps during their last menstruation were reported by 60.9% of participants, of whom 71.5% reported having no resources for pain relief (online supplemental table s2).

Some girls reported completing activities for pay (mean age 16.4 years (SD:2.2)): 16.3% reported doing chores or activities for money in the last month, while 14.6% worked for pay outside the home. For reasons including malaria (56.2%), pregnancy (22.2%), being hit or hurt (2.7%), respiratory infections (2.4%), HIV care (1.2%), STI treatment (0.2%), mental health (0.3%) or other unspecified conditions (14.8%), 64.4% of all the girls reported having accessed healthcare services in the last 6 months.

### Education level prior to school dropout

Experiencing menarche early (<13 years) was associated with dropping out of school prior to completing primary school (mean age: 13.8 years vs 14.3 years, online supplemental table S2, p=0.002, and table 1). Among girls who did not complete primary education, 21.5% experienced menarche early, while only 13.4% of girls who completed primary school experienced menarche before age 13. Young age at first sex, early marriage and higher number of childbirths were also associated with lower educational attainment. Younger age at first sex and at marriage was related to non-completion of primary school (mean age 15.4 years vs 16.2 years and 16.9 years vs 17.4 years, respectively). Higher numbers of live births (1.23 vs 1.08 live births) was associated with not completing primary school. Relative to their peers who completed primary school, girls who did not complete primary school also reported higher rates of risky sexual behaviours such as currently having a partner who was 5+ years older (37.1% vs 25.2%), and lower rates of condom use (49.3% vs 65.1%). They also reported not having a support network to talk to (76.7% vs 84.4%) and higher rates of violence at the hands of a partner (IPV: 21.5% vs 12.7%). These girls less frequently indicated wanting to return to school (34.6% vs 47.0%).

**Table 1:** Factors associated with primary school dropout among out-of-school girls in western Kenya (n=915)

Among all girls	OR (95% CI)	P value	aOR (95% CI)	P value
Age (years)	0.68 (0.61 to 0.76)	**<0.001**	0.64 (0.56 to 0.72)	**<0.001**
SES (poorest 2 vs less poor 3 quintiles)	1.08 (0.82 to 1.42)	0.602	--	--
Not living with a biological parent	1.33 (0.97 to 1.83)	0.079	--	--
Early menarche (<13 years)	1.81 (1.26 to 2.59)	**0.002**	1.50 (1.00 to 2.23)	**0.048**
Talk/visit parents for help and support	0.65 (0.47 to 0.89)	**0.007**	0.61 (0.43 to 0.88)	**0.008**
Talk/visit friends for help and support	0.68 (0.51 to 0.89)	**0.006**	--	--
Talk/visit family or friends for support	0.60 (0.43 to 0.85)	**0.004**	--	--
IPV (any)	1.88 (1.31 to 2.69)	**0.001**	--	--
Sexual IPV	2.32 (1.32 to 4.07)	**0.003**	2.16 (1.08 to 4.34)	**0.030**
Emotional IPV	2.09 (1.36 to 3.22)	**0.001**	--	--
Been hit, slapped, or hurt physically (past 6 months)	1.44 (1.03 to 2.01)	**0.035**	1.48 (1.01 to 2.17)	**0.042**
Used cloth, blanket, rags to manage last period	1.35 (1.02 to 1.80)	**0.039**	1.40 (1.01 to 1.94)	**0.043**
Used tissue, cotton wool to manage last period	0.65 (0.45 to 0.93)	**0.019**	0.61 (0.39 to 0.93)	**0.022**
Had period pain or cramps	0.75 (0.57 to 1.00)	**0.047**	0.68 (0.50 to 0.93)	**0.016**
Wants to return to school (currently)	0.60 (0.45 to 0.79)	**<0.001**	0.47 (0.34 to 0.65)	**<0.001**

Among those with a history of sexual activity	OR (95% CI)	P value	aOR (95% CI)	P value
Age (years)	0.64 (0.56 to 0.73)	**<0.001**	0.66 (0.54 to 0.80)	**<0.001**
SES (poorest 2 vs less poor 3 quintiles)	1.00 (0.74 to 1.35)	0.996	--	--
Not living with a biological parent	1.53 (1.07 to 2.19)	**0.020**	--	--
Early menarche (<13 years)	1.84 (1.25 to 2.71)	**0.002**	1.92 (1.20 to 3.08)	**0.007**
Talk/visit parents for help and support	0.67 (0.47 to 0.94)	**0.022**	--	--
Talk/visit friends for help and support	0.67 (0.50 to 0.91)	**0.009**	--	--
Talk/visit family or friends for support	0.65 (0.45 to 0.95)	**0.026**	--	--
IPV (any)	1.89 (1.31 to 2.72)	**0.001**	--	--
Sexual IPV	2.31 (1.31 to 4.07)	**0.004**	--	--
Emotional IPV	2.09 (1.35 to 3.24)	**0.001**	--	--
Been hit, slapped, or hurt physically (past 6 months)	1.46 (1.02 to 2.10)	**0.037**		
Age at first sex	0.75 (0.63 to 0.89)	**0.001**	0.82 (0.70 to 0.96)	**0.013**
Partner age (5+ years older)	1.75 (1.27 to 2.40)	**0.001**	2.15 (1.44 to 3.21)	**<0.001**
Age at first marriage/cohabitation	0.65 (0.52 to 0.81)	**<0.001**	--	--
Condom use (ever)	0.52 (0.39 to 0.70)	**<0.001**	0.61 (0.42 to 0.90)	**0.012**
Currently pregnant	1.39 (0.98 to 1.97)	0.063	--	--
Number of pregnancies	1.44 (1.05 to 1.97)	**0.025**	--	--
Number of births	1.66 (1.22 to 2.26)	**0.001**	--	--
Used tissue, cotton wool to manage last period	0.64 (0.43 to 0.94)	**0.024**	0.51 (0.31 to 0.85)	**0.009**
Had period pain or cramps	0.73 (0.54 to 0.98)	**0.039**	0.61 (0.41 to 0.90)	**0.012**
Wants to return to school (currently)	0.60 (0.44 to 0.82)	**0.001**	0.47 (0.31 to 0.71)	**<0.001**

Outcome education level: 317 girls had below primary level completion, 598 girls had completed primary but not secondary level. Sample size for adjusted model: 850 for all girls and 584 for those with a history of pregnancy. An OR>1 indicates a increased odds of primary school incompletion, an OR<1 indicates a decreased odds of primary school incompletion. Associations in bold are significant at p<0.05.

aOR, adjusted OR; IPV, intimate partner violence; SES, socioeconomic status.

In the multivariable models, girls who reported experiencing sexual violence from an intimate partner (aOR 2.16, 95% CI 1.08 to 4.34, p=0.030) or any physical violence (aOR 1.48, 95% CI 1.01 to 2.17, p=0.042) had increased odds of dropping out during primary school. Older age (aOR 0.64, 95% CI 0.56 to 0.72, p<0.001) and family support (aOR 0.61, 95% CI 0.43 to 0.88, p=0.008) were associated with girls completing primary school. Girls who reported experiencing menstrual pain (aOR 0.68, 95% CI 0.50 to 0.93, p=0.016) and using tissue and cotton wool to manage their last period (aOR 0.61, 95% CI 0.40 to 0.93, p=0.022) had lower odds of primary school incompletion (table 1), while girls who reported using cloth to manage their menses were more likely to have dropped out prior to completing primary school (aOR 1.40, 95% CI 1.01 to 1.94, p=0.043). Girls who left school prior to completing primary had lower odds of wishing to return to school relative to girls who reached secondary education after adjustment for other co-factors in the multivariable models (aOR 0.47, 95% CI 0.34 to 0.65, p<0.001).

In the multivariable models experiencing menarche early (<13 years) was associated with nearly twice the odds of not completing primary school (1.92, 95% CI 1.20 to 3.08, p=0.007, table 1) for girls who had engaged in sex. For these girls, having a partner who was 5+ years older doubled their odds of not completing primary school (aOR 2.15, 95% CI 1.44 to 3.21, p<0.001); and younger age at first sex was also significantly associated with primary school dropout after adjustment (aOR 0.82, 95% CI 0.70 to 0.96, p=0.013). Condom use was protective to completing primary school in this subgroup (aOR 0.61, 95% CI 0.42 to 0.90, p=0.012).

When looking specifically at factors associated with dropping out of school due to marriage, pregnancy and childcare (online supplemental tableS3), older age (aOR 1.16, 95% CI 1.03 to 1.30, p=0.014), using family planning (aOR 1.67, 95% CI 1.23 to 2.26, p=0.001) and accessing healthcare in the last 6 months (aOR 1.66, 95% CI 1.24 to 2.22, p=0.002) remained associated with dropping out of school after adjustment. Girls who were married or cohabiting and those who worked for pay outside the home were more likely to report having left school due to financial or other reasons (aOR 0.58, 95% CI 0.43 to 0.80, p=0.001 and aOR 0.50, 95% CI 0.33 to 0.75, p=0.001, respectively) (online supplemental tableS3).

### Sexual activity and harassment

Girls who had a history of sexual activity were more likely to not live with their biological parents (75.6% vs 63.2%). They also reported higher rates of sexual harassment (ie, being touched indecently by a boy or man) and feeling scared that they would be sexually assaulted in the last 6 months relative to girls who were not sexually active (18.3% vs 3.2% and 26.1% vs 14.7%, respectively).

In the multivariable models, girls who reported sexual harassment (aOR 4.21, 95% CI 1.71 to 10.42, p=0.002) and were scared that they would be sexually assaulted (aOR 2.04, 95% CI 1.19 to 3.50, p=0.010) had higher odds of experiencing sexual debut (table 2). Talking to friends for help and support (aOR 1.53, 95% CI 1.03 to 2.27, p=0.037) and accessing healthcare services in the last 6 months (aOR 1.82, 95% CI 1.22 to 2.70 p=0.003) was also associated with a history of sexual activity. Being married and older age remained positively correlated with increased odds of sexual activity after adjustment (aOR 12.33, 95% CI 5.21 to 29.20, p<0.001) and (aOR 1.44, 95% CI 1.25 to 1.66, p<0.001), respectively.

**Table 2:** Factors associated with a history of sexual activity (n=915)

Ever sexually active	OR (95% CI)	P value	aOR (95% CI)	P value
Age (years)	1.61 (1.39 to 1.86)	**<0.001**	1.44 (1.25 to 1.66)	**<0.001**
SES (poorest 2 vs less poor 3 quintiles)	1.19 (0.82 to 1.73)	0.365	--	--
Not living with a biological parent	1.80 (1.23 to 2.65)	**0.003**	--	--
Married/cohabiting/widowed	14.17 (6.17 to 32.54)	**<0.001**	12.33 (5.21 to 29.20)	**<0.001**
Talk/visit friends for help and support	1.64 (1.14 to 2.36)	**0.008**	1.53 (1.03 to 2.27)	**0.037**
Indecently touched (past 6 months)	5.04 (2.18 to 11.65)	**<0.001**	4.21 (1.71 to 10.42)	**0.002**
Felt scared would be sexually assaulted (past 6 months).	2.26 (1.36 to 3.76)	**0.005**	2.04 (1.19 to 3.50)	**0.010**
Currently using any FP methods	2.06 (1.32 to 3.22)	**<0.001**	--	--
Used tissue, cotton wool (last period)	1.90 (1.10 to 3.31)	**0.021**	--	--
Menstruation days in last period:				
1–3 days	0.74 (0.51 to 1.10)	0.135	--	--
4–6 days	Ref	--		
7+days	2.39 (1.14 to 5.00)	**0.020**	--	--
Period hindered doing things	1.72 (1.04 to 2.84)	**0.021**	--	--
Used healthcare services (past 6 months)	2.02 (1.40 to 2.92)	**0.002**	1.82 (1.22 to 2.70)	**0.003**

Sexually active in the last 6 months	OR (95% CI)	P value	aOR (95% CI)	P value
Age (years)	1.49 (1.31 to 1.69)	**<0.001**	**--**	--
Age at first marriage/cohabiting (years)	0.68 (0.45 to 1.02)	**0.062**	**--**	--
SES (poorest 2 vs less poor 3 quintiles)	1.42 (1.08 to 1.86)	**0.012**	**--**	--
Not living with a biological parent	2.12 (1.57 to 2.86)	**<0.001**	--	**--**
Married/cohabiting/widowed	9.84 (6.65 to 14.53)	**<0.001**	9.04 (5.64 to 14.5)	**<0.001**
Wanted to resume school at dropout	0.79 (0.60 to 1.04)	**0.091**	**--**	**--**
Wants to resume school (currently)	0.61 (0.47 to 0.80)	**<0.001**	**--**	**--**
Talk/visit friends for help and support	1.39 (1.06 to 1.82)	**0.018**	**--**	**--**
Touched indecently (past 6 months)	2.87 (1.90 to 4.35)	**<0.001**	2.20 (1.35 to 3.58)	**0.002**
Been hit, slapped, or hurt physically (past 6 months)	1.37 (0.97 to 1.94)	**0.074**	**--**	**--**
First sex desired	1.76 (1.29 to 2.42)	**<0.001**	1.55 (1.09 to 2.20)	**0.015**
Ever used condom	1.43 (1.05 to 1.96)	**0.024**	2.41 (1.64 to 3.55)	**<0.001**
Condom use (past 6 months)	2.64 (1.72 to 4.03)	**<0.001**	--	**--**
Ever engaged in transactional sex	2.45 (1.84 to 3.28)	**<0.001**	1.74 (1.20 to 2.51)	**0.003**
Currently using any FP methods	1.92 (1.42 to 2.60)	**<0.001**	--	**--**
Currently using injection BC	0.59 (0.35 to 1.00)	**0.048**	--	**--**
Currently pregnant	2.08 (1.42 to 3.04)	**<0.001**	--	**--**
Ever been pregnant	3.25 (2.36 to 4.48)	**<0.001**	--	**--**
Wanted pregnancy (last pregnancy)	4.02 (2.29 to 7.11)	**<0.001**	**--**	**--**
Did chores for pay/something in return	1.59 (1.07 to 2.36)	**0.022**	**--**	**--**
Used healthcare services (past 6 months)	1.44 (1.09 to 1.90)	**0.010**	**--**	**--**

Outcome: History of sexual activity: 779 girls reported sexual debut; 136 girls had no history of sexual activity. Sample size for adjusted model: 915. Outcome: Sexual activity in past 6 months: 554 girls had been sexually active in the past 6 months; 650 girls had not been sexually active in the past 6 months. Sample size for adjusted model: 779. Associations in bold are significant at p<0.05.

BC, birth control; FP, family planning; SES, socioeconomic status.

Girls who were sexually active in the past 6 months were more likely to live in the poorest SES households (50.9% vs 42.3%) and not live with their biological parents (79.6% vs 71.4%). Sexually active girls also reported higher rates of sexual harassment (27.9% vs 12.2%) and higher rates of engaging in transactional sex (46.8% vs 17.1%).

After adjustment, in the multivariable model girls who reported being married or cohabiting (aOR 9.04, 95% CI 5.64 to 14.5, p<0.001), ever using condoms (aOR 2.41, 95% CI 1.64 to 3.55, p<0.001), engaging in transactional sex (aOR 1.74, 95% CI 1.20 to 2.51, p=0.003) or reporting their first sex as desired (aOR 1.55, 95% CI 1.09 to 2.20, p=0.015) had higher odds of reporting recent sexual activity (table 2). Sexual harassment (aOR 2.20, 95% CI 1.35 to 3.58, p=0.002) was also associated with increased odds of reporting being sexually active in the past 6 months.

### Marital status and pregnancy

Girls who self-reported being married or cohabiting with their partner were more likely to live in the poorest SES households (57.9% vs 42.1%) and voiced less interest in resuming school (26.4% vs 51.4%). These girls reported less family and friend support (76.4% vs 84.5%) and experiencing more IPV (24.2% vs 11.2%) relative to their non-married or cohabiting peers. These girls were more likely to report using family planning (42.0% vs 24.1%) and were less likely to have engaged in transactional sex (29.9% vs 43.3%). Irrespective of marital status, girls who had previously been pregant were more likely to use family planning (35.5% vs 12.5%) but were more likely to report engaging in transactional sex (41.2% vs 30.3%).

In the multivariable models, living in the poorest SES households (aOR 1.98, 95% CI 1.36 to 2.90, p<0.001, table 3) remained significantly associated with being married or cohabiting with a partner after adjustment. Girls who reached menarche prior to age 13 (aOR 0.44, 95% CI 0.27 to 0.74, p=0.002), those who currently desired to return to school (aOR 0.32, 95% CI 0.22 to 0.47, p<0.001), those who had friends and family to turn to for support (aOR 0.36, 95% CI 0.22 to 0.60, p<0.001), and those who reported engaging in transactional sex (aOR 0.29, 95% CI 0.19 to 0.43, p<0.001) had decreased odds of being married or cohabiting with a partner (table 3). Having a partner who was 5+ years older (aOR 1.65, 95% CI 1.10 to 2.47, p=0.016), increasing number of pregnancies (aOR 2.56, 95% CI 1.42 to 4.62, p=0.002) and being on family planning (aOR 1.54, 95% CI 1.04 to 2.28, p=0.032) were associated with being married or cohabiting with a partner after adjustment.

**Table 3:** Factors associated with being married or cohabiting (n=915)

Characteristics	OR (95% CI)	P value	aOR (95% CI)	P value
Age (years)	1.61 (1.37 to 1.90)	**<0.001**	--	--
SES (poorest 2 vs less poor 3 quintiles)	1.89 (1.43 to 2.51)	**<0.001**	1.98 (1.36 to 2.90)	**<0.001**
Early menarche (<13 years)	0.64 (0.43 to 0.95)	**0.028**	0.44 (0.27 to 0.74)	**0.002**
Wanted to resume school after pregnancy	0.46 (0.29 to 0.73)	**0.001**	**--**	**--**
Wants to return to school (currently)	0.34 (0.25 to 0.46)	**<0.001**	0.32 (0.22 to 0.47)	**<0.001**
Talk/visit parents for help and support	0.69 (0.50 to 0.94)	**0.021**	**--**	**--**
Talk to family or friends for support	0.59 (0.42 to 0.84)	**0.003**	0.36 (0.22 to 0.60)	**<0.001**
Ever had sex (consensual or forced)	14.17 (6.17 to 32.54)	**<0.001**	**--**	**--**
Sexually active (past 6 months)	9.83 (6.65 to 14.5)	**<0.001**	9.45 (5.41 to 16.52)	**<0.001**
IPV (any)	2.50 (1.74 to 3.59)	**<0.001**	**--**	**--**
Physical IPV	3.14 (1.99 to 4.94)	**<0.001**	**--**	**--**
Emotional IPV	2.12 (1.38 to 3.27)	**0.001**	**--**	**--**
First sex was with known partner	0.55 (0.33 to 0.91)	**0.019**	**--**	**--**
First sex desired	1.50 (1.11 to 2.02)	**0.008**	**--**	**--**
Partners’ age (5+ years older)	1.38 (1.01 to 1.88)	**0.046**	1.65 (1.10 to 2.47)	**0.016**
Ever engaged in transactional sex	0.56 (0.42 to 0.75)	**<0.001**	0.29 (0.19 to 0.43)	**<0.001**
Currently using any FP methods	2.12 (1.59 to 2.83)	**<0.001**	1.54 (1.04 to 2.28)	**0.032**
Injection	1.60 (1.06 to 2.40)	**0.024**	**--**	**--**
Implant	2.31 (1.62 to 3.30)	**<0.001**		
Currently pregnant	1.82 (1.29 to 2.57)	**0.001**	--	**--**
Ever been pregnant	3.81 (2.53 to 5.74)	**<0.001**	--	**--**
Number of pregnancies	3.90 (2.50 to 6.09)	**<0.001**	2.56 (1.42 to 4.62)	**0.002**
Number of births	4.60 (2.76 to 7.64)	**<0.001**	**--**	**--**
Wanted pregnancy (last pregnancy)	6.64 (4.20 to 10.51)	**<0.001**	**--**	**--**
Used menstrual cups during the last period	0.20 (0.05 to 0.85)	**0.029**	**--**	**--**
Did chores for pay/something in return	1.52 (1.04 to 2.22)	**0.031**	**--**	**--**

Outcome: Married/Cohabiting: 314 girls were married/cohabiting, 601 girls were single/others (not married or cohabiting). Sample size for adjusted model: 689. Associations in bold are significant at p<0.05.

aOR, adjusted OR; BC, birth control; FP, family planning; IPV, intimate partner violence; SES, socioeconomic status.

Reporting a prior pregnancy was positively associated with girl’s age (aOR 1.35, 95% CI 1.19 to 1.53, p<0.001), being married or cohabiting (aOR 2.15, 95% CI 1.37 to 3.39, p=0.001), recently sexually active (aOR 1.98, 95% CI 1.38 to 2.84, p<0.001), on family planning (aOR 3.24, 95% CI 2.06 to 5.11, p<0.001) and accessing healthcare services in the last 6 months (aOR 2.65, 95% CI 1.88 to 3.75, p<0.001) in the multivariable model. After adjustment, working for pay outside home (aOR 0.48, 95% CI 0.30 to 0.76, p=0.002) was associated with lower odds of pregnancy (table 4).

**Table 4:** Factors associated with having been pregnant (n=915)

Among the full cohort of 915 girls	OR (95% CI)	P value	aOR (95% CI)	P value
Age (years)	1.54 (1.35 to 1.75)	**<0.001**	1.35 (1.19 to 1.53)	**<0.001**
SES (poorest 2 vs less poor 3 quintiles)	1.07 (0.78 to 1.47)	0.680	--	--
Not living with a biological parent	1.60 (1.15 to 2.24)	**0.006**	--	--
Married/cohabiting/widowed	3.81 (2.53 to 5.74)	**<0.001**	2.15 (1.37 to 3.39)	**0.001**
Wanted to stop school	0.56 (0.36 to 0.88)	**0.011**		
Sexually active (past 6 months)	3.25 (2.36 to 4.48)	**<0.001**	1.98 (1.38 to 2.84)	**<0.001**
IPV (any)	1.66 (1.03 to 2.67)	**0.037**	--	--
Emotional IPV	1.74 (0.87 to 2.85)	0.065	--	--
Condom use (ever)	0.56 (0.36 to 0.87)	**0.009**	--	--
Condom use (past 6 months)	0.39 (0.26 to 0.59)	**<0.001**	--	--
Ever engaged in transactional sex	1.61 (1.16 to 2.24)	**0.005**		
Currently using any FP methods	3.97 (2.58 to 6.12)	**<0.001**	3.24 (2.06 to 5.11)	**<0.001**
Injection	4.84 (2.21 to 10.59)	**<0.001**	**--**	**--**
Implant	4.42 (2.34 to 8.35)	**<0.001**	**--**	**--**
Used tissue, cotton wool (last period)	1.59 (1.03 to 2.46)	**0.035**	--	--
Menstruation days in last period:				
1–3 days	0.73 (0.52 to 1.01)	0.059	--	**--**
4–6 days	Ref	--	--	--
7+ days	1.82 (1.05 to 3.18)	**0.034**	--	--
Worked for pay outside the home.	0.67 (0.45 to 1.01)	0.058	0.48 (0.30 to 0.76)	**0.002**
Used healthcare services (past 6 months)	2.49 (1.81 to 3.40)	**<0.001**	2.65 (1.88 to 3.75)	**<0.001**

Outcome: History of pregnancy: 707 had a history of pregnancy (ever pregnant+currently pregnant), 208 girls had never been pregnant. Sample size for adjusted model: 915. Associations in bold are significant at p<0.05.

aOR, adjusted OR; FP, family planning; IPV, intimate partner violence; SES, socioeconomic status.

### Support requested for out-of-school girls in western Kenya

**Figure 1:**
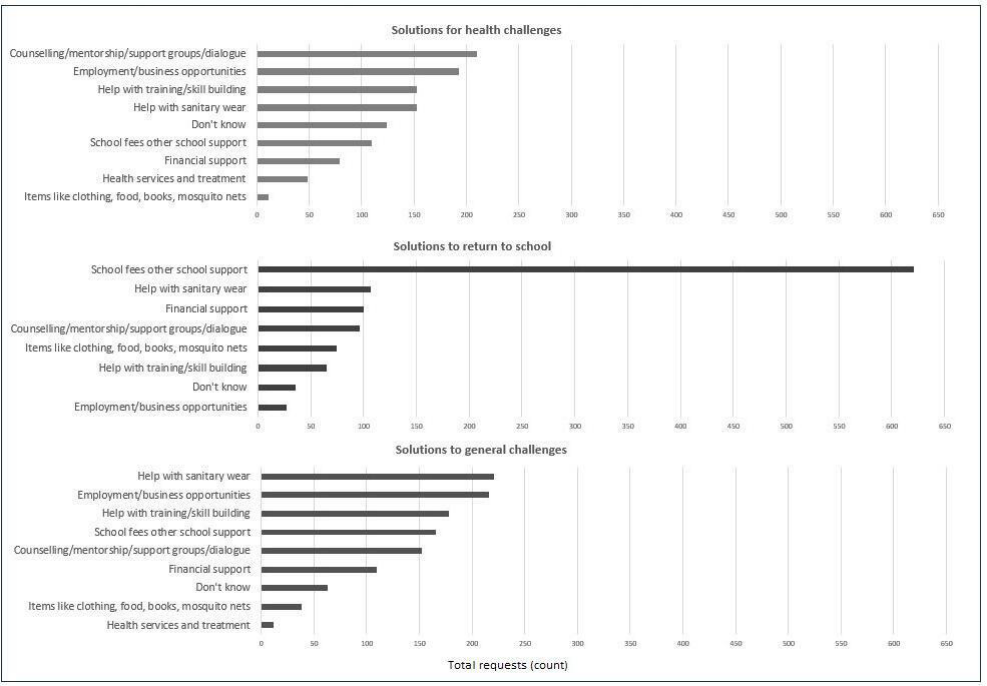
Rank order of types of support girls sought for their health, school and general challenges (n=915). Girls identified solutions they believed would most improve their current health, education, and general challenges. Girls could submit more than one request per category.

Girls identified counselling, mentorship, support groups, and assistance with dialogue with family and partners (22.2%) as the primary support they needed to deal with challenges around health. In this realm, they also identified needing employment and business opportunities (21.1%), period products (16.7%) and vocational training and skill building (16.7%) as solutions to help overcome their challenges related to health. When asked about the support they needed to return to school, 67.9% needed assistance with school fees and other school items, 11.7% sought menstrual products and 10.9% requested financial support. When asked about what solutions may help girls with their general daily challenges, 24.2% of girls requested menstrual products, 23.6% employment and business opportunities, and 19.5% of girls wrote in vocational training and skill building (figure 1).

Across all requests, school fees and school items were most sought (74.4%, n=681), followed by menstrual products (43.5%, n=398), counselling, mentorship, support groups and dialogue (39.3%, n=360), employment and business opportunities (39.0%, n=357) and vocational training and skill building (36.2%, n=331), among other requests (figure 1, online supplemental table S4).

For their health solutions, girls who completed primary school, those who were sexually active and married girls more frequently requested counselling, mentorship and support groups (online supplemental table S5). For schooling solutions, girls who completed primary, those not sexually active and girls who were not married more frequently requested school fees and schooling items. Married girls more frequently requested period products (15% vs 10.0%) relative to those who were not married. For general solutions to daily challenges, sexually active and married girls more frequently requested employment and business opportunities. Requests for period products to mitigate general daily challenges did not differ by participant characteristics.

## Discussion

This paper presents data from 915 out-of-school girls in western Kenya, a sizeable population of hard-to-reach vulnerable adolescent girls.^30^ Over three-quarters of girls did not want to drop out of school, and more than half wanted to resume school after dropping out. Few studies researching adolescents focus on out-of-school girls, despite their greater exposure to early sexual and reproductive health (SRH) risks and vulnerability to long-lasting adverse health and social harms. The overall exposure to risk factors faced by out-of-school girls in western Kenya was notably high.

Our study found an association between reaching menarche early (prior to age 13) and girls’ educational and marital outcomes. One in five girls who reached menarche early did not complete primary level education, and one in three became married during adolescence. Menarche, a girl’s first menstruation, is an important marker of a girl’s physical development and transition into adulthood.^31^ Our findings are aligned with emerging evidence that early menarche is associated with the acquisition and transmission of STIs, early pregnancies and childbirth and sexual violence, indicating it is a driver of adolescent girls’ sexual and reproductive harms.^31^ Our study also found early menarche to be associated with lower educational attainment. While there is growing, although mixed, evidence that menstruation can affect schooling and attendance,^32^,^35^ little attention has been paid to the role of age at menarche mediating this relationship. While this study did not look at these associations in relation to sexually transmitted infections or HIV, a study in Zimbabwe found that, for female scholars, each additional year of schooling resulted in an 11.6 percentage point decrease in the risk of HIV infection,^14^ underscoring the lasting effect factors influencing girls’ schooling can have on their life course.

One-third (34%) of the out-of-school girls in our study were married (mean age: 17.2 years (SD: 1.1)), in line with global statistics that indicate that one in three girls in developing countries are child brides. The Kenyan government ratified the 1990 Convention on the Rights of the Child which set the minimum lawful age of marriage at 18 years. We found that more than half of married girls had not completed primary school, one in five experienced physical and/or emotional violence from their partner and one in three reported not wanting to have sex at sexual debut. Our findings further evidence the negative effects of child marriage on girls’ educational attainment and SRH.^36^,^43^

The majority (89.6%) of girls in our out-of-school population reported having a history of sexual activity, recording mean age at sexual debut at 16.0 years (SD: 1.7). We observed that sexual debut was associated with sexual harassment and an increased fear of sexual assault. These findings are aligned with our previous study among in-school adolescent girls, highlighting that girls in western Kenya are vulnerable to sexual harassment and coercion from a young age.^44^ Although not significant in the multivariable models, our study found that not living with a biological parent was associated with sexual activity among girls, suggesting that the presence of biological parents can be a protective factor against SRH harms. Over one-third of girls reported engaging in transactional sex, which was found to be associated with current sexual activity and pregnancy. One study in Zambia reported a strong relationship between adolescent pregnancy and transactional sex,^45^ finding that exchange sex among adolescents was driven by the need for basic items for themselves and their babies post childbirth, but often resulted in new pregnancies, trapping girls into a cycle of economic instability. Another study in Ghana reported girls engaged in transactional sex to pay for school fees but often ended up pregnant, which then impeded their ability to stay in school.^46^

Our study found that girls who dropped out in primary school were less likely to wish to return to school compared with those who had completed primary. For these girls, resuming school would mean returning to primary school, despite their older ages. Overage enrolment is a pervasive problem in SSA where nearly half of all pupils of lower secondary age are enrolled in primary school.^47^ However, the percentage of overage girls enrolled in school contracts from grade to grade, with studies reporting that simply being overage is a risk factor for dropout as girls progress through education.^47 48^ One reason for this is that the productivity of the child and the opportunity cost of being in school increases with age.^48^ Studies have also documented that for young people, dropout and subsequent grade repetition can be humiliating, with youth reporting fear of being teased and seeing their academic performance as inferior to that of their peers.^49^ In addition to the individual barriers affecting the girl, having a wide age range in the classroom can pose pedagogical problems to an education system that is already overstretched.^50^ Grade curricula may no longer be appropriate for overage students, and the availability of individualised attention and support may be absent.^51^ Some teachers may also not welcome overage students, believing they can change the dynamics in the classroom and be disruptive to classroom learning.^52^ Our findings echo these findings, indicating that being overage, either due to late enrolment, grade repetition or extended time out of school, reduces the likelihood of girls participating at secondary school level, and underscores the need for age-appropriate learning environments, multiage teaching and remedial coursework for older girls who are undereducated.

Our study sought to understand what help girls perceived they needed for their health, schooling and general daily challenges. Three in four girls wanted support to go back to school, including help with school fees and other school items; nearly half of all girls requested provision of period products; one-third of girls requested counselling, support groups and dialogue; one-third requested employment and business opportunities and a similar number asked for vocational training. These requests indicate that most girls do not remain out of school by choice, but rather due to factors beyond their control, and they require assistance to be able to attain better education and health outcomes. Our findings underscore previous evidence indicating that in the absence of appropriate support, school dropout can lead to adolescent pregnancies and other SRH harms and permanently lessen the possibilities of better educational attainment.^53^ Additionally, the girls’ requests for vocational training, employment and support to start small businesses highlight their desire for financial independence as a cornerstone for self-agency and empowerment.

Limitations of our study included the cross-sectional study design, preventing us from establishing temporality to infer causation between the risk factors and our outcomes of interest. It is possible that some outcomes resulted in the selected risk factors, rather than the reverse. Secondly, our study consisted of out-of-school girls from a rural area in Kenya and may not be generalisable to all other adolescent populations, such as school-goers or those living in urban settings, street settings or orphanages. However, we note that most interventions in the literature have given little attention to out-of-school girl adolescent populations living in home care settings and they require further study and targeted solutions. Thirdly, our data were self-reported through surveys, which may be prone to participant response bias, particularly for indicators that evoke a sense of shame for adolescents such as pregnancy or sexual debut. If bias is present, then these indicators would be underreported with the true prevalence greater than reported here. Our study was conducted right after the COVID-19 pandemic, which may have exacerbated some of the study findings.

## Conclusions

Our findings suggest that out-of-school girls face a battery of risks that compound girls’ sexual and reproductive vulnerabilities and have significant negative implications on their educational and SRH outcomes. Early menarche and sexual debut, adolescent pregnancy and child marriage, and the associated childcare responsibilities hinder girls from attaining higher levels of education and impede chances of resuming school after dropping out.^54^ Their vulnerability further exposes them to sexual harassment, IPV and transactional sex, all of which have detrimental effects on girls’ health and well-being. Most girls in our sample did not want to drop out of school and expressed a desire to resume, given the right support with school fees and other school items. Multisectoral interventions that prevent school dropout and support older girls to return to school are essential to safeguard them from life-changing risks and to help improve their educational and health outcomes.^55^ National strategies that are developed to promote adolescent education and health, such as the commitment plans to increase access to menstrual health and hygiene information and products for girls, availability and access to contraceptives and youth-friendly health services, programmes to increase secondary school transition, and school re-entry policies for adolescent mothers should be enforced and routinely measured to strengthen impact. Additionally, tailored economic empowerment programmes and strategies for gender-based violence mitigation and post violence care need to be developed and/or enhanced to advance gender equity and health and education access in this population.

## Supplementary material

10.1136/bmjph-2024-001528 online supplemental file 1

10.1136/bmjph-2024-001528 online supplemental file 2

## Data Availability

Data are available on reasonable request.
