# Supplementary material for: Factors associated with school dropout and sexual and reproductive health: a cross-sectional analysis among out-of-school girls in western Kenya
Source: BMJ Public Health. 2025 Mar 4;3(1):e001528. doi: 10.1136/bmjph-2024-001528 (PMC11883893; doi:10.1136/bmjph-2024-001528)
Supplement: online supplemental file 1 [file bmjph-3-1-s001.pdf]

S1 Annex: Out-of-school girl participant behavioural survey

Variable Name	Question Text	Saved Value
intprompt	### Interviewer prompt: Check if the girl is eligible to participate in this study by asking her the following questions	User entered text
bq1	How old are you?	User entered integer
bq2	Have you ever had a period?	0 No<br>1 Yes
bq3	Are you now currently going to school?	0 No<br>1 Yes
bq4	When did you stop/leave school?	User entered text
y	Enter 4 digit year	User entered integer
m	Enter month number	User entered integer
bq5	Did the girl leave school in the last 5 months (after May 2021)?	0 No<br>1 Yes
intprompt	### Interviewer prompt: If the participant is eligible then continue with the survey:	User entered text
bq8	What is your marital status? Cohabiting means that you are living with your partner but with no formal marriage certificate.	1 Single (not cohabiting)<br>2 Married<br>3 Cohabiting<br>4 Widowed<br>5 Other
bq8_oth	Other, please specify:	User entered text
bq9	Do you remember how old you were when you became married or first started cohabiting with your partner?	0 No<br>1 Yes

bq10	How old were you when you became married or first started cohabiting with your partner?	User entered integer
bq11	Hidden from user	
note	Who are the people you live with now? (Check all that apply)	User entered text
bq11_1	Birth mother	0 No<br>1 Yes
bq11_2	Grandmother	0 No<br>1 Yes
bq11_3	Other female adults	0 No<br>1 Yes
bq11_4	Birth father	0 No<br>1 Yes
bq11_5	Grandfather	0 No<br>1 Yes
bq11_6	Other male adults	0 No<br>1 Yes
bq11_7	Younger brothers	0 No<br>1 Yes
bq11_8	Older brothers	0 No<br>1 Yes
bq11_9	Younger sisters	0 No<br>1 Yes
bq11_10	Older sisters	0 No<br>1 Yes

bq11_11	Own child	0 No<br>1 Yes
bq11_12	Husband/Partner	0 No<br>1 Yes
bq12	Do you have a baby you take care ok?	0 No<br>1 Yes
bq13	Who is the birth mother of this baby?	1 Me<br>2 My partners other wife<br>3 My mother<br>4 A relative in the household<br>5 A relative not in the household<br>6 Other
bq14	Have you ever been to school?	0 No<br>1 Yes
bq15	Please describe why you have never been to school	1 Lack of school fees<br>2 I am an orphan<br>3 I have a deformity<br>4 I need to help at home<br>5 I need to take care of a sibling<br>6 I am not interested<br>7 Religious reasons<br>8 The school is too far from my home<br>9 My parents or relative did not put me in school<br>10 Other
bq15_oth	Other, please specify:	User entered text
skipto27	Hidden from user	
bq16	What level of schooling did you reach by the time you stopped?	1 Started primary did not complete<br>2 Finished Primary

		3 Started secondary did not complete
bq17	In total how many years of schooling did you complete before you stopped?	User entered decimal
bq18	Did you want to stop school?	0 No<br>1 Yes
bq19	Why did you leave school?	1 No money for school fees<br>2 Wanted to work instead<br>3 I got pregnant<br>4 I got married<br>5 I need to take care of a sibling<br>6 I was not interested in continuing school<br>7 I failed<br>8 Other
bq19_oth	Other, please specify:	User entered text
bq20	Did you want to go back to school after pregnancy?	0 No<br>1 Yes
bq21	What was the reason you could not go back?	1 No money for school fees<br>2 School would not let me return back to school<br>3 Needed to work to get income<br>4 Needed to look after baby<br>5 Family refused to let me return to school<br>6 I was teased about having been pregnant<br>7 Other
bq21_oth	Other, please specify:	User entered text
bq22	Did you want to go back to school after you got married?	0 No<br>1 Yes
bq23	What was the reason you could not go back?	

		1 No money for school fees<br>2 School would not let me return back to school<br>3 Needed to work to get income<br>4 Husband/family refused to let me return to school<br>5 Other
bq23_oth	Other, please specify:	User entered text
bq24	Did you want to go back to school after you left?	0 No<br>1 Yes
bq25	What was the reason you could not go back?	1 No money for school fees<br>2 School would not let me return back to school<br>3 Needed to work to get income<br>4 Family refused to let me return to school<br>5 Other
bq25_oth	Other, please specify:	User entered text
bq25.5	Did the COVID-19 pandemic change affect your schooling?	0 No, COVID did not change my ability to go to school<br>1 Yes, I dropped out of school because of COVID<br>2 Yes, I was planning to return to school but could not because of COVID
bq26	Would you like to return and start going to school again now?	0 No<br>1 Yes
bq27	Would you say your general health is:	0 Excellent/good<br>1 Just OK<br>2 Poor
bq28	Are you normally happy at your 'residence'/home?	0 Not happy<br>1 Just OK

		2 Happy
bq29	Do you talk to or visit your parents or trusted family for help and support?	0 Never<br>1 Sometimes<br>2 Regularly
bq29.1	Do you talk to or visit your trusted friends for help and support?	0 Never<br>1 Sometimes<br>2 Regularly
bq30	Do you drink alcohol such as chang'aa, busaa, warage, or other?	0 Never<br>1 Sometimes<br>2 Regularly
bq31	Do you smoke cigarettes?	0 Never<br>1 Sometimes<br>2 Regularly
bq31.1	Do you smoke cannabis such as bhang, njaga, or yugi?	0 Never<br>1 Sometimes<br>2 Regularly
bq32	In the last 6 months, have you been touched indecently by a boy/man?	0 Never<br>1 Just once<br>2 A few times<br>3 Many times
bq32.1	This person or individuals who touched you indecently are:	1 One person who did it once<br>2 One person who does it repeatedly<br>3 Many persons who do it once<br>4 Many persons who do it repeatedly
bq33	In the last 6 months, have you ever felt scared you would be sexually assaulted?	0 Never<br>1 Just once<br>2 A few times

		3	Many times
bq34	In the last 6 months, has anyone hit, slapped, kicked or hurt you physically?	0<br>1<br>2<br>3	Never<br>Just once<br>A few times<br>Many times
bq34_1	Were you injured?	0<br>1	No<br>Yes
bq34_2	Hidden from user		
note	If yes, what was the injury? (Check all that apply)	User entered text	
bq34_2_1	Broken bones	0<br>1	No<br>Yes
bq34_2_2	Bruising	0<br>1	No<br>Yes
bq34_2_3	Loss of consciousness	0<br>1	No<br>Yes
bq34_2_4	Wounds	0<br>1	No<br>Yes
bq34_2_5	Other	0<br>1	No<br>Yes
bq34_2_oth	Other, please specify:	User entered text	
bq35	__In the last 6 months__, have you had sex, or been forced or tricked to have sex with a man or a boy?	0<br>1	No<br>Yes
bq36	Hidden from user		
note	If yes, in the last 6 months did the man or boy physically hurt you in any of the following ways (check all that apply):	User entered text	
bq36_1	Push you, shake you, or throw something at you?	0	No

		1	Yes
bq36_2	Slap you?	0	No
		1	Yes
bq36_3	Twist your arm or pull your hair?	0	No
		1	Yes
bq36_4	Punch you with his fist or with something that could hurt you?	0	No
		1	Yes
bq36_5	Kick you or drag you or beat you up?	0	No
		1	Yes
bq36_6	Try to choke you or burn you on purpose?	0	No
		1	Yes
bq36_7	Threaten or attack you with a knife, gun, or any other weapon?	0	No
		1	Yes
bq37	Hidden from user		
note	__In the last 6 months__ has the man or boy sexually hurt you in any of the following ways (check all that apply):	User entered text	
bq37_1	Ever forced you to have sexual intercourse against your will	0	No
		1	Yes
bq37_2	Ever forced you to perform any other sexual acts against your will	0	No
		1	Yes
bq38	Hidden from user		
bq38	__In the last 6 months__ has the man or boy emotionally hurt you in any of the following ways (check all that apply):	User entered text	
bq38_1	Say or do something to humiliate you in front of others?	0	No
		1	Yes

bq38_2	Threaten to hurt or harm you or someone close to you?	0 No<br>1 Yes
bq38_3	Insult you or make you feel bad about yourself?	0 No<br>1 Yes
bq39	Hidden from user	
note	Due to COVID-19 do you feel that the following has increased, decreased, or stayed the same?	User entered text
bq39_a	The level of violence in your home	1 Increased<br>2 Decreased<br>3 Stayed the same
bq39_b	The level of violence in your village	1 Increased<br>2 Decreased<br>3 Stayed the same
bq39_c	The level of crime in your village	1 Increased<br>2 Decreased<br>3 Stayed the same
bq40	Do boys or men harass you for sex?	0 Never<br>1 A few times<br>2 Many times
bq41	Is this just a few, many or most boys and men?	0 One person<br>1 A couple people<br>2 Lots of different people
bq42	Have you __ever__ had sex with a man or boy?	0 Never<br>1 One time<br>2 A few times<br>3 Many times
bq43	Has a man or boy __ever__ forced or threatened you to make you	0 Never

	have sex?	1 One time<br>2 A few times<br>3 Many times
bq43.1	You stated you are co-habiting or married to a partner, have you ever had sex with this partner?	0 No<br>1 Yes
bq43.2	If no, why have you never had sex with him?	1 My partner has never wanted<br>2 I am too young to have sex<br>3 I am the youngest wife<br>4 I refuse sex<br>5 Other
bq43.2_oth	Other, please specify:	User entered text
skipto61	Hidden from user	
bq44	If you have __ever__ had sex or been forced by a man or boy to have sex, why did it first happen?	1 Got married<br>2 It just happened<br>3 Tricked<br>4 Wanted to prove love<br>5 Curious<br>6 Forced/raped<br>7 Want a child<br>8 Boy wants child<br>9 Drank alcohol<br>10 Boy drank alcohol<br>11 Took drugs<br>12 Boy took drugs<br>13 Offered money<br>14 Given a gift/favour<br>15 Pressure from friends<br>16 Pressure from boy/man<br>17 Pressure from someone else<br>18 Threatened<br>19 Other

bq45	Do you remember how old you were when you __first had sex__ or were forced or threatened into having sex?	0 No<br>1 Yes
bq46	What age were you when you __first had sex__ or were forced or threatened into having sex?	User entered integer
bq47	__The first time__ you had sex (or were forced or threatened to make you have sex with) a boy or man, how old was the man or boy… was he…?	1 Younger than you<br>2 About the same age<br>3 Older than you by less than 5 years<br>4 Older than you by 5-9 years<br>5 Older than you by 10 years or more<br>99 Don't know
bq48	__The first time__ you had sex (or were forced or threatened to make you have sex with) a boy or man, who was the man or boy… was he…?	0 Someone you had never seen before<br>1 Someone you knew
bq49	If you did know him, was he… ?	1 Boyfriend but not living with him<br>2 Husband but not living with him<br>3 Partner/husband after living together<br>4 Relative<br>5 Other person
bq50	__The first time__ you had sex (or were forced or threatened to make you have sex with) a boy or man, did you want to have sex with this man or boy?	0 No<br>1 Yes
bq51	How many boys or men have you had sex with (or been forced or threatened to make you have sex with) __in your life__? It's ok if you can't remember…	User entered integer
bq52	Have you or your partner(s) __ever__ used condoms to delay or avoid a pregnancy or sexually transmitted infection?	0 No<br>1 Yes
bq53	__In the last 6 months__, have you or your partner(s) used condoms to delay or avoid a pregnancy or sexually transmitted infection?	1 Yes<br>0 No: not used
bq54	__In the past 6 months__, how often did the boy or man you were having sex with (or forced or threatened to make you have sex with) use a condom?	0 Never<br>1 Rarely<br>2 Sometimes

		3 Often<br>4 Always
bq55	__The last time__ you had sex (or were forced or threatened to have sex), did the boy or man use a condom?	0 No<br>1 Yes
bq56	How old is the boy or man you have sex with __now__?	1 Younger than you<br>2 About the same age<br>3 Older than you by less than 5 years<br>4 Older than you by 5-9 years<br>5 Older than you by 10 years or more<br>99 Don't know
bq57	Does the boy or man give you something for having sex with him?	0 No<br>1 Yes
bq58	If yes, what?	1 Money<br>2 Food/drink<br>3 House items<br>4 School items<br>5 Help with exams<br>6 Pads for your monthly period<br>7 Less beatings/bad things<br>8 Clothes/things<br>9 Other
bq59	Do you currently have sex with a boy or man who you consider to be your boyfriend, partner, lover or husband?	0 No<br>1 Yes
bq60	How long have you been having sex with this person?	1 Less than 6 months<br>2 6-12 months<br>3 More than 1 year
bq61	Are you __currently__ using any family planning methods?	1 Yes<br>0 No method

bq61.5	Hidden from user	
note	If yes, which family planning method are you using? (Check all that apply)	User entered text
bq61.5_1	Birth control pills	0 No<br>1 Yes
bq61.5_2	Injection	0 No<br>1 Yes
bq61.5_3	Implant	0 No<br>1 Yes
bq61.5_4	Abstinence	0 No<br>1 Yes
bq61.5_5	Other	0 No<br>1 Yes
bq61.5_oth	Other, please specify:	User entered text
bq62	Are you __currently__ pregnant?	0 No<br>1 Yes
bq63	Where in the pregnancy do you think you are?	1 Early (less than 3 months)<br>2 Middle (between 3 months and 6 months)<br>3 Later (more than 6 months pregnant)
bq64	Are you __currently__ trying to get pregnant?	0 No<br>1 Yes
bq65	Have you __ever__ been pregnant?	0 No<br>1 Yes
bq66	How many times have you been pregnant?	User entered integer

bq67	How many babies have you given birth to?	User entered integer
bq68	What happened with the __most recent__ pregnancy?	1 Had a miscarriage<br>2 Aborted/terminated<br>3 Baby born but died<br>4 Baby born alive<br>5 Currently still pregnant<br>6 Other
bq69	If "baby born alive", who does this baby live with?	1 Me<br>2 Not me
bq70	If "not me" then who?	1 My parents<br>2 My sister or brother<br>3 Other family<br>4 The child's father<br>5 The father's family<br>6 Other
bq70_oth	Other, please specify:	User entered text
bq71	Hidden from user	
note	When was this baby born?	User entered text
d	Enter 2 digit day	User entered integer
m	Enter month number	User entered integer
y	Enter 4 digit year	2010 2010<br>2011 2011<br>2012 2012<br>2013 2013<br>2014 2014<br>2015 2015<br>2016 2016<br>2017 2017<br>2018 2018<br>2019 2019<br>2020 2020<br>2021 2021

		2022 | 2022<br>99 | 99
bq72	Have you __ever__ tried to get rid of (abortion / termination) a pregnancy?	0 No<br>1 Yes
bq73	In the current/most recent pregnancy - did you want to become pregnant?	0 No<br>1 Yes
bq74	__In the past 6 weeks__ did you have a period?	0 No<br>1 Yes
bq75	In the past 6 months have you __ever__ used sanitary pads?	0 No<br>1 Yes
bq76	If yes, who has been the main person that provided you with disposable pads (or money to buy pads)?	1 Mother<br>2 Other relative<br>3 Boyfriend/partner<br>4 Person giving money after sex<br>5 Person giving pads after sex<br>6 School<br>7 Community organization<br>8 Church<br>9 Self<br>10 Other
bq76_oth	Other, please specify:	User entered text
bq77	Hidden from user	
note	Now tick any of the persons that have given you pads (or money to buy pads) in the last 6 months?	User entered text
bq77_1	Mother	0 No<br>1 Yes
bq77_2	Other relative	0 No<br>1 Yes

bq77_3	Boyfriend/partner	0 No<br>1 Yes
bq77_4	Person giving money after sex	0 No<br>1 Yes
bq77_5	Person giving pads after sex	0 No<br>1 Yes
bq77_6	Community organisation	0 No<br>1 Yes
bq77_7	Church	0 No<br>1 Yes
bq77_8	Other	0 No<br>1 Yes
bq78	How old were you when you first started your monthly period?	User entered integer
most_recent_period	#### All the next questions are for your most recent period	User entered text
bq79	Did you use any disposable sanitary pads to help manage your period?	1 Yes: entire period<br>2 Yes: part of period<br>0 No
bq80	Did you use any other menstrual wear to help manage your period?	1 Yes: entire period<br>2 Yes: part of period<br>0 No
bq81	Hidden from user	
note	If yes, please specify what __other items__ you used:	User entered text
bq81_1	Cloth, blanket, rags	0 No<br>1 Yes
bq81_2	Reusable pads	0 No<br>[illegible]

		1 Yes
bq81_3	Paper, cardboard	0No<br>1Yes
bq81_4	Menstrual cup	0 No<br>1 Yes
bq81_5	Tissue, cotton wool	0 No<br>1 Yes
bq81_6	Grass, leaves	0 No<br>1 Yes
bq81_7	Other	0 No<br>1 Yes
bq81_oth	Other, please specify:	User entered text
bq81.1	Have you had education or guidance on staying safe and healthy with regards to your period?	0 Never<br>1 A few times<br>2 Many times
bq81.2	Who provided this education or guidance?	1 Parents<br>2 Program<br>3 Church<br>4 Friends<br>5 Internet/TV<br>6 School<br>7 Other
bq81.2_oth	Other, please specify:	User entered text
bq81.3	Have you had education or guidance on staying safe and healthy with regards to sex?	0 Never<br>1 A few times<br>2 Many times

bq81.4	Who provided this education or guidance?	1 Parents<br>2 Program<br>3 Church<br>4 Friends<br>5 Internet/TV<br>6 School<br>7 Other
bq81.4_oth	Other, please specify:	User entered text
bq81.5	Hidden from user	
note	Due to COVID-19, has it been more difficult to:	User entered text
bq81.5_a	Obtain the type of sanitary product you normally use	0 No<br>1 Yes
bq81.5_b	Obtain soap for washing and cleaning one's body	0 No<br>1 Yes
bq81.5_c	Access water for washing or cleaning one's body	0 No<br>1 Yes
bq81.5_d	Have personal privacy for washing, changing, etc.	0 No<br>1 Yes
bq82	How many days did you bleed during your __most recent period__?	User entered integer
bq83	Was it heavy, normal or light?	1 Heavy<br>2 Normal<br>3 Light
bq84	Did your period stop you doing things?	0 No<br>1 Yes
bq85	Hidden from user	
note	If yes, what did you stop doing?	User entered text
bq85_1	House work	0 No

		1 Yes
bq85_2	Employment work	0 No<br>1 Yes
bq85_3	Shamba/farm work	0 No<br>1 Yes
bq85_4	Other work	0 No<br>1 Yes
bq85_5	Other	0 No<br>1 Yes
bq85_oth	Other, please specify:	User entered text
bq86	Did you have pains or cramps?	0 No<br>1 Yes
bq87	If you had pain or cramps how did you deal with this?	0 Had nothing for pain<br>1 Took medicine<br>2 Had other type of pain relief
bq87_oth	Other type of pain relief, please specify:	User entered text
bq88	During your last period, were you at all itchy in your vagina?	0 No<br>1 Yes
bq89	During your last period, have you had any other problems during your period?	0 No<br>1 Yes
bq90	Please describe these problems:	User entered text
bq90_1	Have you experienced stress related to the COVID-19 pandemic?	0 No, no stress at all<br>1 Yes, mild stress such as occasional worries or minor stress-related symptoms such as feeling a little anxious, sad, angry, or mild trouble sleeping

		2 Yes, moderate stress with frequent worries, often feeling anxious, sad, or angry, or some trouble sleeping<br>3 Yes, severe stress with constant worries or feeling extremely anxious, sad, or angry, or frequent trouble sleeping
bq90_2	Have you experienced any stress or discord in your household due to COVID-19?	0 No, none<br>1 Yes, household members occasionally short-tempered with one another; no physical violence<br>2 Yes, household members frequently short-tempered with one another; or children in the home getting in physical fights with one another<br>3 Yes, household members frequently short-tempered with one another and adults in the home throwing things at one another, knocking over furniture, hitting or harming one another
bq90_3	Have you been sick with COVID-19?	0 No<br>1 Yes, I was sick, but effectively managed symptoms at home<br>2 Yes, I was sick, with severe symptoms and required brief hospitalization<br>3 Yes, I was sick, with severe symptoms and required ventilation
bq90_4	Were you diagnosed with a COVID-19 test at a clinic or pharmacy?	0 No<br>1 Yes
bq90_5	How many of your family or friends have been diagnosed with COVID-19?	User entered integer
bq90_6	Have you had a family member or close friend die of COVID-19?	0 No<br>1 Yes
bq90_7	Are there COVID vaccination centers near your home where you can get a COVID vaccine?	1 Yes<br>0 No

		99 Don't know
bq90_7_1	Have you been vaccinated against COVID-19?	0 No<br>1 Yes
bq90_7_2	If yes, did you want to get the COVID-19 vaccine or were you forced to get it?	1 Wanted to get the vaccine<br>2 Did not want to get the vaccine
bq90_7_3	If no, why have you not been vaccinated against COVID-19?	1 Don't want a vaccine<br>2 Distance too far to travel to get one<br>3 Don't know where to get one<br>4 Fear will be too expensive<br>5 Other
bq90_7_3_oth	Other, please specify:	User entered text
bq90_8	Hidden from user	
note	Due to COVID-19, has it been more difficult to:	User entered text
bq90_8_a	Obtain food	1 More difficult to get<br>2 Less difficult to get<br>3 No change in getting food
bq90_8_b	Obtain water	1 More difficult to get<br>2 Less difficult to get<br>3 No change in getting water
bq90_8_c	Obtain cooking oil	1 More difficult to get<br>2 Less difficult to get<br>3 No change in getting cooking oil
bq90_8_d	Obtain clothes	1 More difficult to get<br>2 Less difficult to get<br>3 No change in getting clothes
bq90_8_e	Obtain medicines when you or a family member are ill	1 More difficult to get<br>2 Less difficult to get

		3 No change in getting medicines
bq90_8_f	Seek medical help from a doctor at a clinic or hospital when feeling unwell	1 More difficult to get<br>2 Less difficult to get<br>3 No change in getting medical help
bq90_8_g	Obtain condoms	1 More difficult to get<br>2 Less difficult to get<br>3 No change in getting condoms<br>4 I have not tried to get condoms
bq91	__Yesterday__, how much time did you spend doing household chores, such as cooking, cleaning, laundry, collecting firewood, water?	User entered integer
bq92	Hidden from user	
note	Tick any of the below that you __did last month__:	User entered text
bq92_1	Household chores	0 No<br>1 Yes
bq92_2	Look after siblings	0 No<br>1 Yes
bq92_3	Look after other family	0 No<br>1 Yes
bq92_4	Laundry/mend clothes	0 No<br>1 Yes
bq92_5	Work in the shamba	0 No<br>1 Yes
bq92_6	Fetch water	0 No<br>1 Yes
bq92_7	Help in shop or business	0 No<br>1 Yes

bq92_8	Work in bar	0 No<br>1 Yes
bq92_9	Sex work	0 No<br>1 Yes
bq92_10	Other things	0 No<br>1 Yes
bq93	Has your household chores or work changed due to the COVID-19 pandemic?	1 Yes, I work more hours<br>2 Yes, I work less hours<br>0 No, I work the same number of hours
bq93_1	Do you work for pay outside the home?	0 No<br>1 Yes
bq93_2	What work do you do for pay outside the home?	User entered text
bq94	Have you __ever__ done any chores or activities for which you got paid or were given something in return?	0 No<br>1 Yes
bq95	How old were you the very __first time__ you did any work or activity for pay or favours?	User entered integer
bq95.1	Did you lose your job or source of income due to COVID-19?	0 No<br>1 Yes
bq95.2	Has your household/family income changed due to COVID-19?	1 Yes, it has decreased<br>2 Yes, it has increased<br>3 No, it has stayed the same<br>99 Don't know
bq96	__In the last month__, have you done any chores or activities for which you got paid or were given something in return?	0 No<br>1 Yes
bq97	Hidden from user	

note	If yes, what were you given in return?	User entered text
bq97_1	Money	0 No<br>1 Yes
bq97_2	Personal clothes/things	0 No<br>1 Yes
bq97_3	Clothes	0 No<br>1 Yes
bq97_4	Food/drink	0 No<br>1 Yes
bq97_5	Soap	0 No<br>1 Yes
bq97_6	Book	0 No<br>1 Yes
bq97_7	Phone	0 No<br>1 Yes
bq97_8	Pads for monthly period	0 No<br>1 Yes
bq97_9	House things	0 No<br>1 Yes
bq97_10	Favours	0 No<br>1 Yes
bq97_11	Less beatings	0 No<br>1 Yes

bq97_12	Other	
bq97_oth	Other, please specify:	User entered text
bq97.5	Aside from housework, have you done any work in the __last seven days__?	0 No<br>1 Yes
bq98	Have you ever had sex in order to pay for things, or get favours?	0 No<br>1 Yes
bq99	Hidden from user	
note	If yes, what have you been given in return?	User entered text
bq99_1	Money	0 No<br>1 Yes
bq99_2	Personal clothes/things	0 No<br>1 Yes
bq99_3	Clothes	0 No<br>1 Yes
bq99_4	Food/drink	0 No<br>1 Yes
bq99_5	Soap	0 No<br>1 Yes
bq99_6	Book	0 No<br>1 Yes
bq99_7	Phone	0 No<br>1 Yes
bq99_8	Pads for monthly period	0 No<br>1 Yes

bq99_9	House things	0 No<br>1 Yes
bq99_10	Favours	0 No<br>1 Yes
bq99_11	Less beatings	0 No<br>1 Yes
bq99_12	Other	0 No<br>1 Yes
bq99.1	Due to COVID-19, have you had sex with someone in order to receive money or something else?	0 No<br>1 Yes
bq99.2	Hidden from user	
note	What did the boy or man give you in exchange for having sex with him? (Please check all that apply)	User entered text
bq99.2_1	Money	0 No<br>1 Yes
bq99.2_2	Personal clothes/things	0 No<br>1 Yes
bq99.2_3	Clothes	0 No<br>1 Yes
bq99.2_4	Food/drink	0 No<br>1 Yes
bq99.2_5	Soap	0 No<br>1 Yes
bq99.2_6	Book	

bq99.2_7	Phone	0 No<br>1 Yes
bq99.2_8	Pads for monthly period	0 No<br>1 Yes
bq99.2_9	House things	0 No<br>1 Yes
bq99.2_10	Favours	0 No<br>1 Yes
bq99.2_11	Other	0 No<br>1 Yes
bq100	In the last month did anyone give you money, gifts, or presents?	0 No<br>1 Yes
bq101	If yes, what did they give you in the last month?	1 Money<br>2 Personal clothes/things<br>3 Clothes<br>4 Food/drink<br>5 Soap<br>6 Book<br>7 Phone<br>8 Pads for monthly period<br>9 House things<br>10 Favours<br>11 Other
bq102	Why did they give you the money/gift/present?	1 Birthday<br>2 For work done

		3 After sex<br>4 After helping<br>5 To say sorry<br>6 Other
bq103	Hidden from user	
note	In the last six months have you experienced any of the following?	User entered text
bq103_1	Accident	0 No<br>1 Yes
bq103_2	Drunk alcohol	0 No<br>1 Yes
bq103_3	Smoked cigarettes	0 No<br>1 Yes
bq103_4	Got high from a drug	0 No<br>1 Yes
bq103_5	Had sex	0 No<br>1 Yes
bq103_6	Been bullied	0 No<br>1 Yes
bq103_7	Been hit or hurt	0 No<br>1 Yes
bq103_8	Illness	0 No<br>1 Yes
bq103_9	Went to health facility	0 No<br>1 Yes
bq104	In the past 6 months did you go or need to use health care services?	

bq105	Who was the health provider / health providers?	1 Local health facility<br>2 Local health care worker in community<br>3 Local nyamwera / birth attendant<br>4 Local traditional healer<br>5 County hospital (Siaya, Kisumu)<br>6 Other
bq105_oth	Other, please specify:	User entered text
bq106	Please describe what the health problem was?	1 Malaria<br>2 HIV<br>3 Pneumonia or other respiratory infection<br>4 Sexually transmitted infection<br>5 Pregnancy<br>6 Been hit or hurt<br>7 Other
bq106_oth	Other, please specify:	User entered text
bq107	For girls like yourself who are not in-school, what is the most difficult health problem girls have?	User entered text
bq108	How can people help support out-of-school girls with this?	User entered text
bq109	What other items or solutions can be given to girls out-of-school to help them?	User entered text
bq110	What things can be given to you and other girls to help them go back into schooling?	User entered text
