# Supplementary material for: Factors associated with school dropout and sexual and reproductive health: a cross-sectional analysis among out-of-school girls in western Kenya
Source: BMJ Public Health. 2025 Mar 4;3(1):e001528. doi: 10.1136/bmjph-2024-001528 (PMC11883893; doi:10.1136/bmjph-2024-001528)
Supplement: online supplemental file 2 [file bmjph-3-1-s002.docx]

**S2 Annex: Supplemental Tables**

| **Variable selection** | **Education level: Among all study girls** | **Education level: Among those with a history of sexual activity** | **Dropped out due to marriage, pregnancy,**  **childcare** | **Ever-sexually active** | **Sexually active in the last 6months** | **Married or cohabiting** | **Prior Pregnancy** |
| --- | --- | --- | --- | --- | --- | --- | --- |
|  | # of times selected | # of times selected | # of times selected | # of times selected | # of times selected | # of times selected | # of times selected |
| Age (yrs) | **1000** | **997** | **754** | **999** | 386 | **924** | **999** |
| SES | 269 | 112 | 119 | 222 | 202 | **431** | 281 |
| Not living with a biological parent | 566 | 395 | -- | 118 | 188 | -- | 140 |
| Early menarche (<13yrs) | **584** | **713** | -- | -- | -- | **396** | -- |
| Married/cohabiting | -- | -- | **854** | **991** | **1000** | -- | **928** |
| Wanted to stop school | -- | -- | 633 | -- | -- | -- | 686 |
| Wanted to resume school at time of dropout | -- | -- | -- | -- | 138 | 178 | -- |
| Wants to return to school (currently) | **999** | **973** | **638** | -- | 124 | **904** | -- |
| Talk/visit parents for help and support | **681** | 297 | -- | -- | -- | -- | -- |
| Talk/visit friends for help and support | 210 | 167 | -- | **420** | 172 | -- | -- |
| Talk/visit family or friends for support | -- | -- | -- | -- | -- | **600** | -- |
| Any IPV | -- | -- | 362 | -- | -- | -- | 245 |
| Physical IPV | -- | -- | 239 | -- | -- | 142 | -- |
| Sexual IPV | **630** | 421 | -- | -- | -- | -- | -- |
| Emotional IPV | 371 | 162 | -- | -- | -- | 269 | 229 |
| Indecently touched by a man (past 6 months) | -- | -- | -- | **959** | **696** | -- | -- |
| Felt scared would be sexually assaulted (last 6 months). | -- | -- | -- | **886** | -- | -- | -- |
| Been hit, slapped, or hurt physically (past 6 months) | **477** | 376 | -- | -- | 115 | -- | -- |
| Age at first sex | -- | **758** | -- | -- | -- | -- | -- |
| First sex was with known partner | -- | -- | -- | -- | -- | 201 | -- |
| Wanted to have sex the first time | -- | -- | -- | -- | **603** | 186 | -- |
| Partners age (5+ years older) | -- | **862** | -- | -- | -- | **351** | -- |
| Sexually active in past 6 months | -- | -- | -- | -- | -- | **991** | **951** |
| Ever engaged in transactional sex | -- | -- | -- | -- | **607** | **1000** | 334 |
| Condom use (ever) | -- | **700** | -- | -- | 263 | -- | -- |
| Condom use (past 6 months) | -- | -- | -- | -- | **966** | -- | -- |
| Currently using **any** FP methods | -- | -- | **937** | 310 | 468 | **329** | **999** |
| Currently pregnant | -- | 107 | -- | -- | 203 | 178 | -- |
| Number of pregnancies | -- | 693 | -- | -- | -- | **933** | -- |
| Wanted pregnancy (last pregnancy) | -- | -- | -- | -- | 525 |  |  |
| Used cloth, blanket, rags to manage last period | **558** | -- | -- | -- | -- | -- | -- |
| Used tissue, cotton wool to manage last period | **672** | **810** | -- | 561 | -- | -- | 319 |
| Had period pain or cramps | **708** | **818** | -- | -- | -- | -- | -- |
| Period hindered doing things | -- | -- | -- | 304 | -- | -- | -- |
| Menstruation days in last period | -- | -- | 367 | 719 | -- | -- | 534 |
| Worked for pay outside the home. | -- | -- | **973** | -- | -- | -- | **894** |
| Did chores for pay/something in return | -- | -- | -- | -- | 266 | 303 | -- |
| Used health care services (past 6 months) | -- | -- | **984** | **903** | 144 | -- | **1000** |

**S1 Table: Stepwise model variable selection check via ‘swboot’ bootstrap replications.**

Legend: Footnote: Bootstrapped replications using STATA swboot were performed to validate variable selection of stepwise model. Bold indicates those retained in the stepwise models.

**S2 Table: General characteristics of out-of-school girls in western Kenya (n=915)**

| **PARTICIPANT CHARACTERISTICS** | **Total** | **Education: Minimal / none** | **Completed Primary school+** | **Dropout due to (pregnancy/ marriage/ childcare)** | **Dropout due to (school fees/ need to work/income)** | **Sexual debut (Ever sexually active)** | **Currently sexually active (< 6 months)** | **History of marriage**  **(ever married)** | **History of pregnancy**  **(ever pregnant)** | **Childcare responsibilities** |
| --- | --- | --- | --- | --- | --- | --- | --- | --- | --- | --- |
|  | (N = 915) | (n = 317) | (n = 598) | (n = 381) | (n = 381) | (n = 779) | (n = 554) | (n = 314) | (n = 707) | (n = 600) |
| Age (in years) median (IQR) | 18.6 (17.5-19.4) | 18.3 (16.8-19.3) | 18.7 (17.9-19.4) | 18.7 (17.6-19.4) | 18.6 (17.5-19.3) | 18.7 (17.8-19.4) | 18.8 (18.0-19.4) | 19.0 (18.3-19.5) | 18.8 (17.9-19.4) | 18.8 (18.0-19.4) |
| Age (in years) mean (SD) | 18.3 (1.3) | 18.0 (1.5) | 18.5 (1.1) | 18.4 (1.3) | 18.3 (1.2) | 18.5 (1.2) | 18.6 (1.2) | 18.8 (1.1) | 18.5 (1.2) | 18.6 (1.2) |
| Age at menarche (in years) mean (SD)^a(^ | 14.1 (1.6) | 13.8 (1.7) | 14.3 (1.5) | 14.0 (1.6) | 14.2 (1.6) | 14.1 (1.6) | 14.1 (1.6) | 14.3 (1.6) | 14.1 (1.6) | 14.1 (1.6) |
| SES (poorest 2 vs less poor 3 quintiles) | 421 (47.5%) | 151 (48.7%) | 270 (46.9%) | 173 (46.4%) | 177 (48.4%) | 364 (48.1%) | 273 (50.9%) | 175 (57.9%) | 329 (47.9%) | 276 (47.4%) |
| Not living with a biological parent | 675 (73.8%) | 245 (77.3%) | 430 (71.9%) | 277 (72.7%) | 286 (75.1%) | 589 (75.6%) | 441 (79.6%) | 303 (96.5%) | 537 (76.0%) | 453 (75.5%) |
| **SCHOOLING HISTORY** |  |  |  |  |  |  |  |  |  |  |
| Ever been to school? | 896 (97.9%) | 298 (94.0%) | 598 (100.0%) | 381 (100.0%) | 381 (100.0%) | 803 (97.9%) | 541 (97.7%) | 307 (97.8%) | 694 (98.2%) | 588 (98.0%) |
| Completed primary school | 598 (65.4%) | 0 (0.0%) | 598 (100.0%) | 253 (66.4%) | 293 (76.9%) | 535 (65.2%) | 363 (65.5%) | 196 (62.4%) | 454 (64.2%) | 384 (64.0%) |
| Did not want to stop school | 791 (88.3%) | 257 (86.2%) | 534 (89.3%) | 349 (91.6%) | 352 (92.4%) | 676 (88.7%) | 480 (88.7%) | 264 (86.0%) | 623 (89.8%) | 525 (89.3%) |
| Reason for dropping out: |  |  |  |  |  |  |  |  |  |  |
| Pregnancy/Marriage/Childcare^(b)^ | 381 (42.5%) | 128 (43.0%) | 253 (42.3%) | 381 (100.0%) | 0 (0.0%) | 354 (46.5%) | 228 (42.1%) | 111 (36.2%) | 381 (54.9%) | 321 (54.6%) |
| School fees/wanting to work ^(b)^ | 381 (42.5%) | 88 (29.5%) | 293 (49.0%) | 0 (0.0%) | 381 (100.0%) | 309 (40.6%) | 239 (44.2%) | 147 (47.9%) | 236 (34.0%) | 200 (34.0%) |
| Not interested in school | 50 (5.6%) | 32 (10.7%) | 18 (3.0%) | 0 (0.0%) | 0 (0.0%) | 38 (5.0%) | 24 (4.4%) | 21 (6.8%) | 34 (4.9%) | 31 (5.3%) |
| Other unspecified reasons | 20 (2.2%) | 7 (2.3%) | 13 (2.2%) | 0 (0.0%) | 0 (0.0%) | 16 (2.1%) | 14 (2.6%) | 6 (2.0%) | 8 (1.2%) | 9 (1.5%) |
| Failed at school | 37 (4.1%) | 24 (8.1%) | 13 (2.2%) | 0 (0.0%) | 0 (0.0%) | 26 (3.4%) | 21 (3.9%) | 13 (4.2%) | 22 (3.2%) | 16 (2.7%) |
| Illness | 27 (3.0%) | 19 (6.4%) | 8 (1.3%) | 0 (0.0%) | 0 (0.0%) | 19 (2.5%) | 15 (2.8%) | 9 (2.9%) | 13 (1.9%) | 11 (1.9%) |
| Wanted to return to school after dropping out | 499 (55.7%) | 65 (52.0%) | 157 (64.6%) | 222 (60.3%) | 0 (0.0%) | 222 (60.3%) | 289 (53.4%) | 48 (46.6%) | 222 (60.3%) | 189 (61.2%) |
| Reason could not return to school |  |  |  |  |  |  |  |  |  |  |
| Childcare/refused entry/teased ^(c)^ | 259 (70.4%) | 101 (80.8%) | 158 (65.0%) | 259 (70.4%) | 0 (0.0%) | 259 (70.4%) | 155 (70.8%) | 73 (70.9%) | 259 (70.4%) | 219 (70.9%) |
| No school fees/Needed income ^(c)^ | 109 (29.6%) | 24 (19.2%) | 85 (35.0%) | 109 (29.6%) | 381 (100%) | 99 (29.0%) | 104 (69.8%) | 30 (29.1%) | 109 (29.6%) | 90 (29.1%) |
| Would like to return to school now ^(b)^ | 384 (42.9%) | 103 (34.6%) | 281 (47.0%) | 183 (48.0%) | 171 (44.9%) | 336 (41.8%) | 206 (38.1%) | 81 (26.4%) | 297 (42.8%) | 251 (42.7%) |
| **SEXUAL HISTORY** |  |  |  |  |  |  |  |  |  |  |
| Married/cohabitating or widowed | 314 (34.3%) | 118 (37.2%) | 196 (32.8%) | 111 (29.1%) | 147 (38.6%) | 313 (38.2%) | 280 (50.5%) | 314 (100.0%) | 283 (40.0%) | 238 (39.7%) |
| Age at first marriage (in years) mean (SD)^(d)^ | 17.2 (1.1) | 16.9 (1.3) | 17.4 (1.0) | 17.4 (1.1) | 17.3 (1.0) | 17.2 (1.1) | 17.2 (1.2) | 17.2 (1.1) | 17.3 (1.1) | 17.3 (1.1) |
| Ever had sex | 779 (85.1%) | 285 (89.9%) | 535 (89.5%) | 381 (100.0%) | 309 (81.1%) | 820 (100.0%) | 554 (100.0%) | 313 (99.7%) | 707 (100.0%) | 592 (98.7%) |
| Currently sexually active (< 6 months) | 554 (60.5%) | 115 (36.3%) | 150 (25.1%) | 92 (24.1%) | 117 (30.7%) | 265 (32.3%) | 554 (100.0%) | 137 (43.6%) | 221 (31.3%) | 170 (28.3%) |
| First sex desired ^(e)^ | 471 (60.5%) | 161 (59.2%) | 310 (61.1%) | 214 (60.5%) | 63 (16.5%) | 471 (60.5%) | 357 (64.4%) | 204 (66.2%) | 407 (61.1%) | 342 (61.4%) |
| Age at first sex (in years) mean (SD) ^(f)^ | 16.0 (1.7) | 15.4 (1.9) | 16.2 (1.6) | 16.0 (1.3) | 16.0 (2.0) | 16.0 (1.7) | 15.9 (1.9) | 15.9 (1.9) | 15.9 (1.7) | 16.0 (1.6) |
| Knew first sexual partner | 711 (91.3%) | 246 (90.4%) | 465 (91.7%) | 324 (91.5%) | 281 (90.9%) | 711 (91.3%) | 506 (91.3%) | 272 (88.3%) | 608 (91.3%) | 514 (92.3%) |
| Who was he? ^(g)^ |  |  |  |  |  |  |  |  |  |  |
| Boyfriend/husband but not living together | 605 (85.1%) | 200 (81.3%) | 405 (87.1%) | 285 (88.0%) | 234 (83.3%) | 605 (85.1%) | 422 (83.4%) | 194 (71.3%) | 514 (84.5%) | 437 (85.0%) |
| Boyfriend/husband after living together | 75 (10.5%) | 32 (13.0%) | 43 (9.2%) | 27 (8.3%) | 34 (12.1%) | 75 (10.5%) | 68 (13.4%) | 70 (25.7%) | 69 (11.3%) | 58 (11.3%) |
| Relative | 12 (1.7%) | 5 (2.0%) | 7 (1.5%) | 4 (1.2%) | 6 (2.1%) | 12 (1.7%) | 7 (1.4%) | 2 (0.7%) | 8 (1.3%) | 5 (1.0%) |
| Other | 19 (2.7%) | 9 (3.7%) | 10 (2.2%) | 8 (2.5%) | 7 (2.5%) | 19 (2.7%) | 9 (1.8%) | 6 (2.2%) | 17 (2.8%) | 14 (2.7%) |
| Condom use (ever)^(e)^ | 464 (59.6%) | 134 (49.3%) | 330 (65.1%) | 203 (57.3%) | 195 (63.1%) | 464 (59.6%) | 344 (62.1%) | 177 (57.5%) | 384 (57.7%) | 328 (58.9%) |
| Condom use (past 6 months) ^(h)^ | 289 (62.3%) | 80 (59.7%) | 209 (63.3%) | 110 (54.2%) | 131 (67.2%) | 289 (62.3%) | 235 (68.3%) | 111 (62.7%) | 225 (58.6%) | 189 (57.6%) |
| Partner 5+ years older ^(e)^ | 229 (29.4%) | 101 (37.1%) | 128 (25.2%) | 106 (29.9%) | 82 (26.5%) | 229 (29.4%) | 155 (28.0%) | 103 (33.4%) | 200 (30.0%) | 176 (31.6%) |
| Ever engaged in transactional sex | 354 (38.7%) | 118 (37.2%) | 236 (39.5%) | 155 (40.7%) | 145 (38.1%) | 354 (43.2%) | 259 (46.8%) | 94 (29.9%) | 291 (41.2%) | 244 (40.7%) |
| Currently pregnant? | 161 (17.6%) | 66 (20.8%) | 95 (15.9%) | 74 (19.4%) | 57 (15.0%) | 161 (19.6%) | 119 (21.5%) | 74 (23.6%) | 161 (22.8%) | 57 (9.5%) |
| Ever been pregnant? | 707 (77.3%) | 253 (79.8%) | 454 (75.9%) | 381 (100.0%) | 236 (61.9%) | 707 (86.2%) | 474 (85.6%) | 283 (90.1%) | 707 (100.0%) | 575 (95.8%) |
| Number of pregnancies mean (SD) ^(i)^ | 0.97 (0.71) | 1.07 (0.71) | 0.91 (0.70) | 1.20 (0.59) | 0.79 (0.70) | 1.06 (0.67) | 1.09 (0.66) | 1.29 (0.73) | 1.22 (0.58) | 1.23 (0.60) |
| Number of babies mean (SD) ^(j)^ | 1.14 (0.42) | 1.23 (0.49) | 1.08 (0.37) | 1.15 (0.37) | 1.12 (0.47) | 1.14 (0.42) | 1.17 (0.46) | 1.28 (0.53) | 1.14 (0.42) | 1.16 (0.40) |
| Ever tried to get rid of a pregnancy | 28 (4.1%) | 11 (4.5%) | 17 (3.9%) | 14 (3.9%) | 9 (3.8%) | 28 (4.1%) | 20 (4.3%) | 11 (4.0%) | 28 (4.1%) | 24 (4.3%) |
| Wanted pregnancy (last pregnancy) ^(i)^ | 119 (17.4%) | 50 (20.4%) | 69 (15.7%) | 39 (10.9%) | 59 (25.0%) | 119 (17.4%) | 104 (22.5%) | 91 (32.9%) | 119 (17.4%) | 88 (15.9%) |
| Currently using a family planning method ^(k)^ | 285 (31.2%) | 102 (32.2%) | 183 (30.6%) | 137 (36.0%) | 106 (27.8%) | 258 (33.1%) | 206 (37.2%) | 134 (42.7%) | 259 (36.6%) | 255 (42.5%) |
| Currently using implant BC | 151 (52.1%) | 55 (52.4%) | 96 (51.9%) | 71 (51.1%) | 7 (6.5%) | 138 (52.5%) | 106 (51.5%) | 77 (57.5%) | 140 (53.2%) | 142 (54.8%) |
| Currently using injection BC | 109 (37.6%) | 36 (34.3%) | 73 (39.5%) | 58 (41.7%) | 38 (35.2%) | 96 (36.5%) | 70 (34.0%) | 48 (35.8%) | 102 (38.8%) | 101 (39.0%) |
| Currently using BC pills | 16 (5.5%) | 9 (8.6%) | 7 (3.8%) | 6 (4.3%) | 7 (6.5%) | 14 (5.3%) | 10 (4.9%) | 3 (2.2%) | 14 (5.3%) | 10 (3.9%) |
| Other FP methods | 16 (5.5%) | 6 (5.7%) | 10 (5.4%) | 5 (3.6%) | 8 (7.4%) | 16 (6.1%) | 15 (7.3%) | 5 (3.7%) | 10 (3.8%) | 10 (3.9%) |
| **VIOLENCE** |  |  |  |  |  |  |  |  |  |  |
| IPV (any) ^(l)^ | 144 (15.7%) | 68 (21.5%) | 76 (12.7%) | 48 (12.6%) | 63 (16.5%) | 144 (17.6%) | 144 (26.0%) | 76 (24.2%) | 121 (17.1%) | 91 (15.2%) |
| Physical IPV ^(l)^ | 86 (9.4%) | 35 (11.0%) | 51 (8.5%) | 28 (7.3%) | 45 (11.8%) | 86 (10.5%) | 86 (15.5%) | 51 (16.2%) | 72 (10.2%) | 54 (9.0%) |
| Sexual IPV ^(l)^ | 52 (5.7%) | 28 (8.8%) | 24 (4.0%) | 20 (5.2%) | 18 (4.7%) | 52 (6.3%) | 52 (9.4%) | 20 (6.4%) | 42 (5.9%) | 33 (5.5%) |
| Emotional IPV ^(l)^ | 93 (10.2%) | 47 (14.8%) | 46 (7.7%) | 35 (9.2%) | 35 (9.2%) | 93 (11.3%) | 93 (16.8%) | 47 (15.0%) | 79 (11.2%) | 59 (9.8%) |
| Normally happy at your 'residence'/home | 738 (80.7%) | 254 (80.1%) | 484 (80.9%) | 302 (79.3%) | 307 (80.6%) | 658 (80.2%) | 449 (81.0%) | 258 (82.2%) | 562 (79.5%) | 473 (78.8%) |
| Talk/visit parents or family for help and support | 708 (77.4%) | 229 (72.2%) | 479 (80.1%) | 294 (77.2%) | 295 (77.4%) | 638 (77.8%) | 427 (77.1%) | 229 (72.9%) | 552 (78.1%) | 472 (78.7%) |
| Talk/visit friends for help and support | 558 (61.0%) | 174 (54.9%) | 384 (64.2%) | 232 (60.9%) | 239 (62.7%) | 509 (62.1%) | 355 (64.1%) | 193 (61.5%) | 437 (61.8%) | 382 (63.7%) |
| Talk to family or friends for support | 748 (81.7%) | 243 (76.7%) | 505 (84.4%) | 311 (81.6%) | 314 (82.4%) | 674 (82.2%) | 453 (81.8%) | 240 (76.4%) | 584 (82.6%) | 501 (83.5%) |
| Indecently touched (past 6 months) | 153 (16.7%) | 46 (14.5%) | 107 (17.9%) | 55 (14.4%) | 64 (16.8%) | 150 (18.3%) | 121 (21.8%) | 55 (17.5%) | 115 (16.3%) | 90 (15.0%) |
| Scared of being sexually assaulted (<6 months) | 228 (24.9%) | 85 (26.8%) | 143 (23.9%) | 93 (24.4%) | 82 (21.5%) | 214 (26.1%) | 139 (25.1%) | 69 (22.0%) | 183 (25.9%) | 152 (25.3%) |
| Was hit, slapped, hurt physically (<6 months) | 176 (19.2%) | 73 (23.0%) | 103 (17.2%) | 65 (17.1%) | 80 (21.0%) | 162 (19.8%) | 117 (21.1%) | 66 (21.0%) | 131 (18.5%) | 102 (17.0%) |
| **MENSTRUATION** |  |  |  |  |  |  |  |  |  |  |
| Early menarche <13yrs | 148 (17.1%) | 68 (22.8%) | 80 (14.0%) | 66 (18.1%) | 54 (15.0%) | 127 (17.1%) | 91 (17.2%) | 39 (13.1%) | 115 (17.1%) | 95 (16.8%) |
| Used disposable sanitary pads (last period) | 804 (87.9%) | 277 (87.4%) | 527 (88.2%) | 343 (90%) | 332 (87.2%) | 719 (87.6%) | 484 (87.4%) | 276 (87.9%) | 623 (88.1%) | 528 (88%) |
| Used other menstrual wear (last period) | 462 (50.5%) | 174 (54.9%) | 288 (48.2%) | 193 (50.7%) | 186 (48.8%) | 393 (50.5%) | 270 (48.7%) | 152 (48.4%) | 358 (50.6%) | 298 (49.6%) |
| Cloth, blanket, rags | 303 (33.1%) | 119 (37.5%) | 184 (30.8%) | 114 (29.9%) | 125 (32.8%) | 278 (33.9%) | 188 (33.9%) | 108 (34.4%) | 239 (33.8%) | 203 (33.8%) |
| Tissue, cotton wool | 174 (19.0%) | 47 (14.8%) | 127 (21.2%) | 78 (20.5%) | 64 (16.8%) | 164 (20.0%) | 115 (20.8%) | 56 (17.8%) | 145 (20.5%) | 128 (21.3%) |
| Reusable pads | 168 (18.4%) | 59 (18.6%) | 109 (18.2%) | 67 (17.6%) | 75 (19.7%) | 152 (18.5%) | 99 (17.9%) | 50 (15.9%) | 125 (17.7%) | 109 (18.2%) |
| Paper, cardboard | 24 (2.6%) | 8 (2.5%) | 16 (2.7%) | 9 (2.4%) | 6 (1.6%) | 22 (2.7%) | 15 (2.7%) | 7 (2.2%) | 19 (2.7%) | 17 (2.8%) |
| Menstrual cup | 21 (2.3%) | 6 (1.9%) | 15 (2.5%) | 11 (2.9%) | 7 (1.8%) | 21 (2.6%) | 10 (1.8%) | 2 (0.6%) | 17 (2.4%) | 13 (2.2%) |
| Grass, leaves | 15 (1.6%) | 6 (1.9%) | 9 (1.5%) | 5 (1.3%) | 5 (1.3%) | 14 (1.7%) | 7 (1.3%) | 4 (1.3%) | 11 (1.6%) | 6 (1.0%) |
| Period duration |  |  |  |  |  |  |  |  |  |  |
| 1-3 days | 407 (45.0%) | 145 (46.5%) | 262 (44.2%) | 155 (41.2%) | 178 (47.0%) | 332 (43.2%) | 243 (44.2%) | 141 (45.5%) | 295 (42.3%) | 247 (41.6%) |
| 4-6 days | 361 (39.9%) | 126 (40.4%) | 235 (39.6%) | 161 (42.8%) | 144 (38.0%) | 309 (40.2%) | 218 (39.6%) | 112 (36.1%) | 283 (40.6%) | 242 (40.7%) |
| 7+ days | 137 (15.1%) | 41 (13.1%) | 96 (16.2%) | 60 (16.0%) | 57 (15.0%) | 128 (16.6%) | 89 (16.2%) | 57 (18.4%) | 119 (17.1%) | 105 (17.7%) |
| Period severity |  |  |  |  |  |  |  |  |  |  |
| Heavy | 252 (27.5%) | 90 (28.4%) | 162 (27.1%) | 107 (28.1%) | 95 (24.9%) | 227 (27.7%) | 155 (28.0%) | 91 (29.0%) | 199 (28.1%) | 167 (27.8%) |
| Normal | 513 (56.1%) | 165 (52.1%) | 348 (58.2%) | 207 (54.3%) | 238 (62.5%) | 468 (57.1%) | 318 (57.4%) | 170 (54.1%) | 398 (56.3%) | 336 (56.0%) |
| Light | 150 (16.4%) | 62 (19.6%) | 88 (14.7%) | 67 (17.6%) | 48 (12.6%) | 125 (15.2%) | 81 (14.6%) | 53 (16.9%) | 110 (15.6%) | 97 (16.2%) |
| Had period-related pain or cramps | 557 (60.9%) | 179 (56.5%) | 378 (63.2%) | 224 (58.8%) | 247 (64.8%) | 503 (61.3%) | 339 (61.2%) | 198 (63.1%) | 426 (60.3%) | 351 (58.5%) |
| Had nothing to manage period pain | 398 (71.5%) | 141 (78.8%) | 257 (68.0%) | 163 (72.8%) | 173 (70.0%) | 356 (70.8%) | 102 (30.1%) | 138 (69.7%) | 307 (72.1%) | 249 (70.9%) |
| **WORK HISTORY / OTHER** |  |  |  |  |  |  |  |  |  |  |
| Age at first work for pay (in years) mean (SD) ^(n)^ | 16.4 (2.2) | 16.1 (2.0) | 16.5 (2.3) | 16.5 (2.4) | 16.3 (2.3) | 16.4 (2.2) | 16.4 (2.1) | 16.5 (2.1) | 16.4 (2.4) | 16.5 (2.2) |
| Worked for pay outside the home. | 134 (14.6%) | 48 (15.1%) | 86 (14.4%) | 40 (10.5%) | 67 (17.6%) | 119 (14.5%) | 87 (15.7%) | 45 (14.3%) | 95 (13.4%) | 89 (14.8%) |
| Done chores or activities for pay (past month) | 149 (16.3%) | 46 (14.5%) | 103 (17.2%) | 54 (14.2%) | 60 (19.1%) | 133 (16.2%) | 89 (16.1%) | 44 (14.0%) | 108 (15.3%) | 97 (16.2%) |
| Used health care services (past 6 months) | 589 (64.4%) | 195 (61.5%) | 394 (65.9%) | 269 (70.6%) | 65 (17.1%) | 543 (66.2%) | 375 (67.7%) | 212 (67.5%) | 490 (69.3%) | 404 (67.3%) |

Legend: Denominators vary for some indicators: (a) 868 girls knew their age at first menstrual period; (b)-896 girls had ever been to school; (c)-368 girls who dropped out due to pregnancy; (d)272 girls who remember the age when they got married; (e)- 779 girls who had ever had sex or forced or threatened to make them have sex; (f)-604 who remember the age when they first had sex or forced/threatened to have sex; (g)-711 girls who knew the person they first had sex with or forced or threatened to make them have sex; (h)-554 who had been sexually active in the last 6 months; (i)-707 girls with a pregnancy history; (j)-589 girls with a history of childbirth; (k)-290 girls using some form of family planning method; (l)-265 who had a sexual partner in past 6 months; (m)-176 girls who’ve been hit, slapped, kicked or hurt physically in the past 6 months; (n)-256 girls who knew the ages when they worked for pay or favours. Abbreviations: BC: Birth control, FP: Family planning, IPV: Intimate Partner Violence.

**S3 Table: Factors associated with dropping out of school due to marriage, pregnancy and childcare responsibilities when compared to those who dropped out due to financial/other reasons**

| **Characteristics** | **OR (95% CI)** | **P value** | **aOR (95% CI)** | **P value** |
| --- | --- | --- | --- | --- |
| Age (yrs) | 1.11 (1.00-1.24) | **0.052** | 1.15 (1.03-1.30) | **0.014** |
| SES (poorest 2 vs less poor 3 quintiles) | 0.92 (0.70-1.20) | 0.521 | -- | -- |
| Married/Cohabiting/Widowed | 0.67 (0.50-0.89) | **0.006** | 0.58 (0.43-0.80) | **0.001** |
| Wanted to stop school | 0.56 (0.36-0.86) | **0.009** | -- | -- |
| Wants to return to school (currently) | 1.44 (1.10-1.89) | **0.007** | 1.37 (1.04-1.82) | **0.028** |
| IPV (any) | 0.69 (0.47-1.01) | **0.055** | -- | -- |
| Physical IPV | 0.65 (0.40-1.05) | **0.075** | -- | -- |
| Condom use (past 6 months) | 0.54 (0.37-0.79) | **0.002** | -- | -- |
| Current use of any FP methods | 1.49 (1.12-1.99) | **0.006** | 1.67 (1.23-2.26) | **0.001** |
| Wanted pregnancy (last pregnancy) | 0.36 (0.24-0.55) | **<0.001** | -- | -- |
| Menstruation days in last period: |  |  |  |  |
| 1-3 days | 0.75 (0.56-1.00) | **0.049** | -- | -- |
| 4-6 days | ref | -- | -- | -- |
| 7+ days | 0.93 (0.63-1.39) | **0.728** | -- | -- |
| Worked for pay outside the home. | 0.56 (0.38-0.84) | **0.005** | 0.50 (0.33-0.75) | **0.001** |
| Used health care services in past 6 months | 1.60 (1.21-2.12) | **0.001** | 1.66 (1.24-2.22) | **0.002** |

Legend: Outcome reason for dropout: 381 girls dropped out due to pregnancy, marriage and/or childcare responsibilities, 515 girls dropped out due to financial and other personal reasons. Sample size for adjusted model: 896. An OR > 1 indicates a increased odds of dropping out due to pregnancy, childcare, or marriage vs financial and other reasons. Abbreviations: SES: Socio-economic status IPV: intimate partner violence; FP: family planning

|  | **Health**  **Freq (%)** | **School**  **Freq (%)** | **General**  **Freq (%)** | **Total girls**  **Freq (%)** |  |
| --- | --- | --- | --- | --- | --- |
| School fees/school items | 110 (12.0%) | 621 (67.9%) | 166 (18.1%) | 681 (74.4%) |  |
| Help with pads and/or period products | 153 (16.7%) | 107 (11.7%) | 221 (24.2%) | 398 (43.5%) |  |
| Counselling/mentorship/support group/life skills/supported dialogue | 210 (23.0%) | 96 (10.5%) | 152 (16.6%) | 360 (39.3%) |  |
| Employment or business opportunities | 193 (21.1%) | 27 (3.0%) | 216 (23.6%) | 357 (39.0%) |  |
| Training/vocational education | 153 (16.7%) | 65 (7.1%) | 178 (19.5%) | 331(36.2%) |  |
| Financial support | 79 (8.6%) | 100 (10.9%) | 110 (12.0%) | 247 (27.0%) |  |
| Uncategorized/Don’t know | 124 (13.6%) | 36 (3.9%) | 63 (6.9%) | 173 (18.9%) |  |
| Items like clothing, food, books, etc | 11 (1.2%) | 72 (8.2%) | 38 (4.2%) | 90 (9.8%) |  |
| Health services | 48 (5.2%) | 0 (0.0%) | 12 (1.3%) | 58 (6.3%) |  |

**S4 Table: Support girls requested for their health, school, and general challenges (n=915)**

| **Support asked for by out-of-school girls in Western Kenya.** | **Total** | **Education: Minimal/ none** | **Completed Primary school +** | **Dropout (pregnancy/ marriage/ childcare)** | **Dropout (school fees/need to work)** | **Ever sexually active** | **Currently sexually active (past 6 months)** | **Ever married** | **Ever pregnant** |
| --- | --- | --- | --- | --- | --- | --- | --- | --- | --- |
|  | **(N = 915)** | **(N = 317)** | **(N = 598)** | **(N = 381)** | **(N = 381)** | **(N = 779)** | **(N = 554)** | **(N = 314)** | **(N = 707)** |
| **Health Solutions** |  |  |  |  |  |  |  |  |  |
| Counselling/mentorship/support group/life skills | 210 (23.0%) | 62 (19.6%) | 148 (24.7%) | 88 (23.1%) | 87 (22.8%) | 183 (23.5%) | 146 (26.4%) | 83 (26.4%) | 168 (23.8%) |
| Employment or business opportunities | 193 (21.1%) | 76 (24.0%) | 117 (19.6%) | 81 (21.3%) | 78 (20.5%) | 164 (21.1%) | 119 (21.5%) | 80 (25.5%) | 155 (21.9%) |
| Vocational training/skill building | 153 (16.7%) | 52 (16.4%) | 101 (16.9%) | 64 (16.8%) | 67 (17.6%) | 130 (16.7%) | 89 (16.1%) | 45 (14.3%) | 122 (17.3%) |
| Help with pads or period products | 153 (16.7%) | 53 (16.7%) | 100 (16.7%) | 76 (19.9%) | 58 (15.2%) | 132 (16.9%) | 87 (15.7%) | 51 (16.2%) | 127 (18.0%) |
| School fees/school items | 110 (12.0%) | 34 (10.7%) | 76 (12.7%) | 46 (12.1%) | 49 (12.9%) | 95 (12.2%) | 66 (11.9%) | 28 (8.9%) | 83 (11.7%) |
|  |  |  |  |  |  |  |  |  |  |
| **Schooling Solutions** |  |  |  |  |  |  |  |  |  |
| School fees/school items/support back to school | 621 (67.9%) | 192 (60.6%) | 429 (71.7%) | 272 (71.4%) | 262 (68.8%) | 525 (67.4%) | 359 (64.8%) | 189 (60.2%) | 481 (68.0%) |
| Help with pads or period products | 107 (11.7%) | 44 (13.9%) | 63 (10.5%) | 40 (10.5%) | 45 (11.8%) | 94 (12.1%) | 70 (12.6%) | 47 (15.0%) | 85 (12.0%) |
| Financial support | 100 (10.9%) | 42 (13.2%) | 58 (9.7%) | 31 (8.1%) | 48 (12.6%) | 88 (11.3%) | 64 (11.6%) | 42 (13.4%) | 78 (11.0%) |
| Support with counselling/mentorship/support group/life skills | 96 (10.5%) | 31 (9.8%) | 65 (10.9%) | 51 (13.4%) | 24 (6.3%) | 81 (10.4%) | 66 (11.9%) | 35 (11.1%) | 74 (10.5%) |
| Food/books/personal effects/clothing | 72 (7.9%) | 26 (8.2%) | 46 (7.7%) | 31 (8.1%) | 35 (9.2%) | 62 (8.0%) | 44 (7.9%) | 28 (8.9%) | 58 (8.2%) |
|  |  |  |  |  |  |  |  |  |  |
| **General Solutions** |  |  |  |  |  |  |  |  |  |
| Help with pads or period products | 221 (24.2%) | 77 (24.3%) | 144 (24.1%) | 96 (25.2%) | 84 (22.0%) | 183 (23.5%) | 131 (23.6%) | 73 (23.2%) | 169 (23.9%) |
| Employment or business opportunities | 216 (23.6%) | 77 (24.3%) | 139 (23.2%) | 91 (23.9%) | 91 (23.9%) | 196 (25.2%) | 147 (26.5%) | 89 (28.3%) | 185 (26.2%) |
| Vocational training/skill building | 178 (19.5%) | 58 (18.3%) | 120 (20.1%) | 71 (18.6%) | 79 (20.7%) | 151 (19.4%) | 96 (17.3%) | 57 (18.2%) | 138 (19.5%) |
| School fees/school items/support back to school | 166 (18.1%) | 50 (15.8%) | 116 (19.4%) | 69 (18.1%) | 79 (20.7%) | 139 (17.8%) | 98 (17.7%) | 47 (15.0%) | 122 (17.3%) |
| Counselling/mentorship/support group/life skills | 152 (16.6%) | 59 (18.6%) | 93 (15.6%) | 78 (20.5%) | 44 (11.5%) | 135 (17.3%) | 101 (18.2%) | 55 (17.5%) | 128 (18.1%) |

**S5 Table: Support asked for by out-of-school girls in western Kenya (n=915)**
